# Molecular Phylogeny of the Astrophorida (Porifera, *Demospongiae* ^p^) Reveals an Unexpected High Level of Spicule Homoplasy

**DOI:** 10.1371/journal.pone.0018318

**Published:** 2011-04-08

**Authors:** Paco Cárdenas, Joana R. Xavier, Julie Reveillaud, Christoffer Schander, Hans Tore Rapp

**Affiliations:** 1 Department of Biology, University of Bergen, Bergen, Norway; 2 CIBIO – Research Centre for Biodiversity and Genetic Resources, CIBIO-Azores, Biology Department, University of the Azores, Azores, Portugal; 3 CEAB – Center for Advanced Studies of Blanes (CSIC), Blanes, Spain; 4 Marine Biology Section, Biology Department, Ghent University, Ghent, Belgium; 5 CeMoFE, Center for Molecular Phylogeny and Evolution, Ghent, Belgium; 6 Centre for Geobiology, University of Bergen, Bergen, Norway; Biodiversity Insitute of Ontario - University of Guelph, Canada

## Abstract

**Background:**

The Astrophorida (Porifera, *Demospongiae* ^p^) is geographically and bathymetrically widely distributed. *Systema Porifera* currently includes five families in this order: Ancorinidae, Calthropellidae, Geodiidae, Pachastrellidae and Thrombidae. To date, molecular phylogenetic studies including Astrophorida species are scarce and offer limited sampling. Phylogenetic relationships within this order are therefore for the most part unknown and hypotheses based on morphology largely untested. Astrophorida taxa have very diverse spicule sets that make them a model of choice to investigate spicule evolution.

**Methodology/Principal Findings:**

With a sampling of 153 specimens (9 families, 29 genera, 89 species) covering the deep- and shallow-waters worldwide, this work presents the first comprehensive molecular phylogeny of the Astrophorida, using a cytochrome *c* oxidase subunit I (COI) gene partial sequence and the 5′ end terminal part of the 28S rDNA gene (C1-D2 domains). The resulting tree suggested that i) the Astrophorida included some lithistid families and some Alectonidae species, ii) the sub-orders Euastrophorida and Streptosclerophorida were both polyphyletic, iii) the Geodiidae, the Ancorinidae and the Pachastrellidae were not monophyletic, iv) the Calthropellidae was part of the Geodiidae clade (*Calthropella* at least), and finally that v) many genera were polyphyletic (*Ecionemia*, *Erylus*, *Poecillastra*, *Penares*, *Rhabdastrella*, *Stelletta* and *Vulcanella*).

**Conclusion:**

The Astrophorida is a larger order than previously considered, comprising ca. 820 species. Based on these results, we propose new classifications for the Astrophorida using both the classical rank-based nomenclature (i.e., Linnaean classification) and the phylogenetic nomenclature following the *PhyloCode*, independent of taxonomic rank. A key to the Astrophorida families, sub-families and genera *incertae sedis* is also included. Incongruences between our molecular tree and the current classification can be explained by the banality of convergent evolution and secondary loss in spicule evolution. These processes have taken place many times, in all the major clades, for megascleres and microscleres.

## Introduction

**Figure 1:**
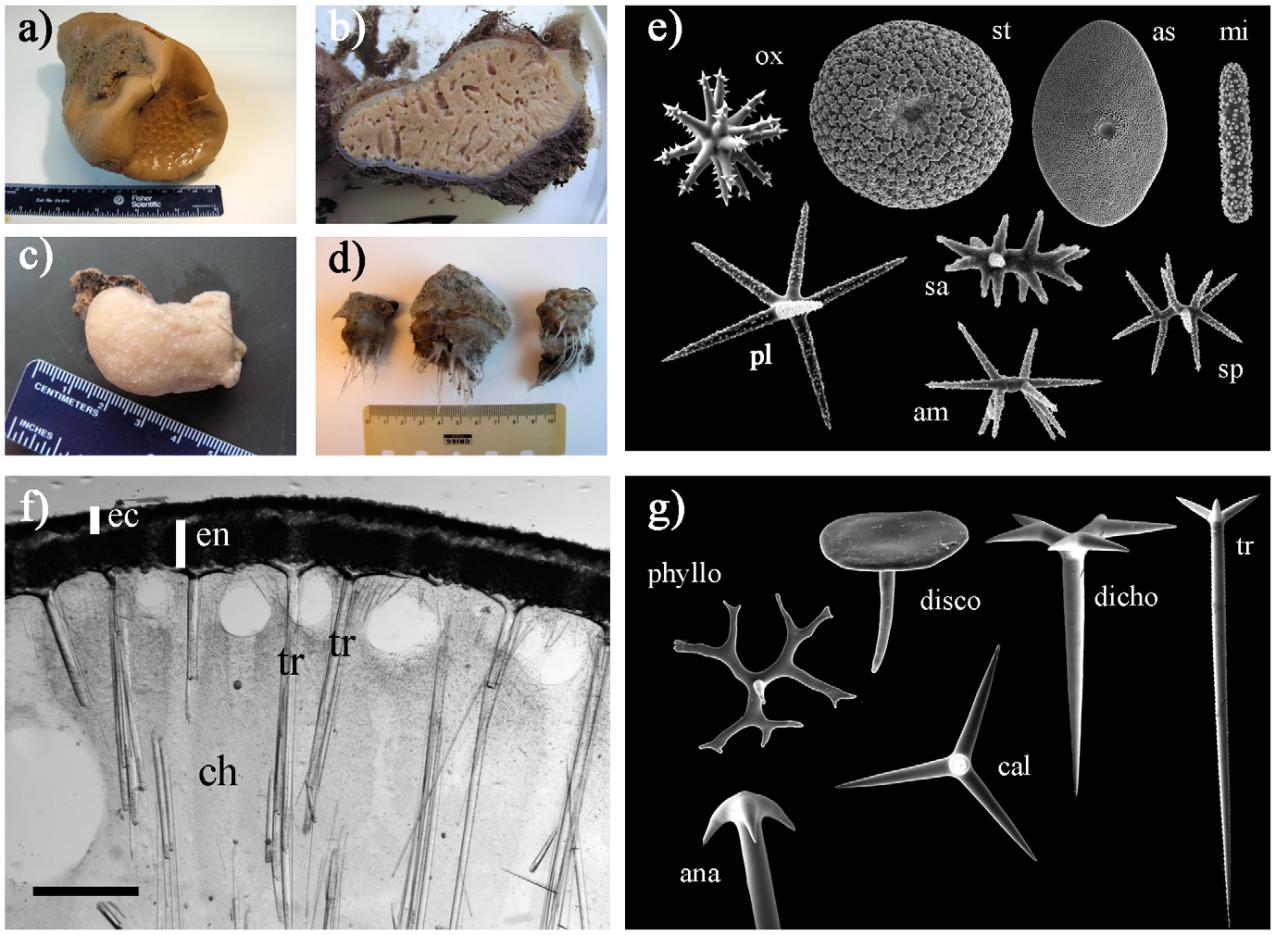
Presentation of the Astrophorida morphology. (a–d) A few Astrophorida species. (a): *Geodia phlegraei* (Geodiidae) collected in the Denmark Strait. Uniporal oscules are on the top surface. (b): Cross-section of a *Stelletta raphidiophora* (Ancorinidae) collected on the ‘Schultz Massive’ seamount (Greenland Sea) (ZMBN 85223). The grayish thick cortex is clearly visible. Specimen is 13 cm in diameter. (c): *Calthropella geodioides* (Calthropellidae) collected South of the Azores (ZMAPOR 21659). (d): *Thenea valdiviae* (Pachastrellidae) collected on the Norwegian coast. (e): Characteristic Astrophorida microscleres. *ox* – oxyaster of *Geodia papyracea* (diameter: 23 µm); *st* – sterraster of *Geodia barretti* (diameter: 80 µm); *as* – aspidaster of *Erylus expletus* (length: 330 µm); *mi* – microrhabd of *Pachymatisma normani* (length: 20 µm); *pl* – plesiaster of *Poecillastra compressa* (diameter: 37 µm); *sa* – sanidaster of *Stryphnus raratriaenus*; *am* – amphiaster of *Characella pachastrelloides* (length: 18 µm); *sp* – spiraster of *Thenea levis* (length: 23 µm). (f): cross-section of the cortex of *Geodia barretti* showing the skeleton organization. *ec* – ectocortex made of a thin layer of strongylaster and microxeas. *en* – endocortex made of a thick layer of sterrasters. *ch* – choanosome. *tr* – triaene supporting the cortex. Scale: 1 mm. (g): Characteristic Astrophorida megascleres. *cal* – calthrop of *Pachastrella* sp. from Norway (actine length: 100 µm); *tr* – long-shafted triaene of *Stelletta* sp. from Panama (rhabdome length: 850 µm); *dicho* – dichotriaene of *Characella pachastrelloides* (rhabdome length: 500 µm); ana – cladome of anatriaene of *Geodia tumulosa* from Panama (clad length: 24 µm); *disco* – discotriaene of *Discodermia polymorpha* (disc diameter: 180 µm) (photo: A. Pisera); *phyllo* – phyllotriaene of *Theonella* sp. (cladome: 730 µm) (photo: A. Pisera).

*Demospongiae* ^p^ Sollas, 1885 [Borchiellini et al., 2004] make up 85% of all living sponges, and is today subdivided in 13 extant orders. Based on molecular results, *Demospongiae* ^p^ are subdivided in four clades: G1/*Keratosa* ^p^ [Borchiellini et al., 2004], G2/*Myxospongiae* ^p^ [Borchiellini et al., 2004], G3/Haplosclerida and G4/Democlavia [1], [2]. The Astrophorida Sollas, 1888 are found within the Democlavia clade and represent one of the few sponge orders to have been consistently and with strong support, shown to be monophyletic [1], [3], [4], [5]. The Astrophorida is geographically and bathymetrically widely distributed around the world, and represent around 660 extant species (van Soest *et al.* 2010[6]; this study). In tropical and parts of warm temperate waters Astrophorida species are common at quite shallow depths, while in boreal/antiboreal and Arctic/Antarctic waters they are usually deep-water species. Astrophorida species have colonized hard- as well as soft-bottoms from various depths. In gravely hard-bottom habitats on the outer shelf and upper slope, Astrophorida can dominate ecosystems in terms of abundance and biomass forming sponge grounds [7], [8]. Astrophorida species display a wide array of external morphologies (massive to thin encrusting, subspherical-, fan-, cup- or irregularly-shaped) and external colors (Fig. 1a–d), and they range in size from a few millimeters to more than a meter in diameter. There is no single morphological synapomorphy of the Astrophorida. They are nonetheless well characterized by the simultaneous presence of star-shaped microscleres (small spicules called ‘asters’) and tetractinal megascleres (large spicules called ‘triaenes’) (Fig. 1e–g). Star-shaped microscleres may be euasters — asters in which the rays radiate from a central point (e.g. oxyasters, strongylasters, spherasters, sterrasters) or streptasters — asters in which the rays proceed from an axis that can be straight (amphiasters) or spiral (e.g. spirasters, metasters, plesiasters). According to the latest major revision of the Astrophorida [9], five families are included: Ancorinidae Schmidt, 1870, Calthropellidae Lendenfeld, 1907, Geodiidae Gray, 1867, Pachastrellidae Carter, 1875, and Thrombidae, Sollas, 1888. Thirty-eight genera and two subgenera are currently distributed in those families. In an effort to incorporate some lithistids in the Astrophorida, the sub-orders Euastrophorida Reid, 1963 (Astrophorida with euasters) and Streptosclerophorida Dendy, 1924 (Astrophorida/lithistids with streptasters) were erected [10], [11], but in spite of molecular evidence confirming their incorporation within the Astrophorida [5], [12], [13], lithistids have been kept apart in the *Systema Porifera* [14]. Other taxa such as the boring sponges *Alectona* and *Neamphius* have also been suggested to be derived Astrophorida species, based on morphological [15], molecular [16] and larval data [17], [18], but they are still considered to belong to the order Hadromerida in the *Systema Porifera* [19].

The Astrophorida is an order with one of the most diverse spicule repertoire among the *Demospongiae* ^p^. For example, *Geodia barretti* (Geodiidae, Astrophorida) has up to ten different spicule types while *Halichondria panicea* (Halichondriidae, Halichondrida) has only one. This spicule diversity within the Astrophorida is ideal to trace spicule evolution and thereby evaluate the importance of homoplasy in this group. Homoplasy (convergent evolution and secondary loss) has always been acknowledged by sponge taxonomists and phylogeneticists but few studies have been able to show to what extent these evolutionary processes occur in sponges, due to the paucity of spicule types and morphological characters. Secondary loss has been particularly difficult to reveal in morphological studies and molecular studies of species with too few spicule types. Meanwhile, the paraphyly and polyphyly of many sponge orders in *Demospongiae* ^p^ and Calcarea (e.g. Haplosclerida, Halichondrida, Clathrinida, Murrayonida) in molecular phylogenetic studies clearly suggest that the evolution of spicules may be more intricate than currently thought [3], [4], [20], [21], [22], [23].

To date, the most complete molecular phylogenetic study focusing on the Astrophorida is based on ten species belonging to six families, including two species of lithistids [24]. Other *Demospongiae* ^p^ molecular phylogenies include only three to six species of Astrophorida [1], [4]. With over 660 species of Astrophorida described worldwide [6], needless to say that phylogenetic relationships within this order are for the most part unknown and hypotheses based on morphology largely untested. And, since Astrophorida families might not be monophyletic [24], any Astrophorida phylogenetic study needs to have the broadest sampling as possible, from the five Astrophorida families as well as from putative Astrophorida (lithistids, *Alectona*, *Neamphius*). With a sampling of 153 specimens (9 families, 89 species) covering the deep- and shallow-waters of the Atlantic, Pacific, Indian, and Southern Ocean, the overall aim of this work was to present the first comprehensive molecular phylogeny of the Astrophorida. More specifically, the first aim of this study was to test the monophyly i) of the Euastrophorida/Streptosclerophorida sub-orders and ii) of the Astrophorida families and genera. Our second aim was to revise the taxonomy of this order using both the classical rank-based nomenclature (i.e. Linnaean classification) and the phylogenetic nomenclature following the *PhyloCode*, independent of taxonomic rank. To be clear, names established under the *PhyloCode* are always in italics and will be identified with the symbol ‘p’ (e.g. *Demospongiae* ^p^). Authors of *PhyloCode* names are between square brackets (e.g. *Demospongiae* ^p^ Sollas, 1885 [Borchiellini et al., 2004]). Finally, our third aim was to investigate the evolution of Astrophorida megascleres and microscleres in order to evaluate the importance of homoplastic spicule characters in this order.

## Materials and Methods

### Ethics statement

This study has been approved by the University of Bergen through the acceptance of a Ph.D. project proposal.

### Sponge sampling

Most of our collecting was done in the Northeast Atlantic. Sampling in the Korsfjord (60°10′N, 05°10′E), Langenuen (59°53′N, 05°31′E) and the Hjeltefjord (60°24′N, 05°05′E) (Western Norway, south of Bergen) were carried out using a triangular dredge and a bottom trawl between 40 and 500 meters (between the years 2005 and 2009). Southern Norway samples (58°13′N, 08°35′E) were dredged during the BIOSKAG 2006 cruise. Northern Norway samples were collected during the *Polarstern* ARK-XXII/1a 2007 cruise with large boxcores and the *Jago* manned-submersible. Localities sampled were Sotbakken (70°45′N, 18°40′E), Røst reef (67°30′N, 9°24′E) and Trænadjupet (66°58′N, 11°7′E). Greenland Sea samples were collected on the “The Schultz Massive” seamount (73°47′N, 07°40′E) during the BIODEEP 2007 and H2DEEP 2008 cruises using the ROV *Bathysaurus XL*. Samples from Bocas del Toro (9°20′N, 82°15′E, Panama, Atlantic), Berlengas Islands (39°24′N, 09°30′W, Portugal) and the Azores Islands were collected by snorkeling/diving. The Gorringe Bank (36°31′N, 11°34′W) specimens were collected by diving during Luso Expedição 2006 [25]. Samples from deep-water coral reefs off Cape Santa Maria di Leuca (Ionian Sea, Apulian Plateau, 39°33′N, 18°26′E) were collected with the ROV *Victor* and an Usnel core during the ‘Ifremer MEDECO 2007’ cruise. Samples of the seamounts Southern of the Azores were collected in the course of the campaigns EMEPC-G3-2007/2008 of the Task Group for the Extension of the Continental Shelf (EMEPC, Portugal) employing the ROV *Luso*. Other samples were kindly provided by different institutions and scientists (cf. Acknowledgments). Hologenophores — a sample or preparation of the same individual organism as the study organism [26] — were preserved in 95% ethanol and stored at room temperature at the Bergen Museum. Species, voucher numbers, Genbank accession numbers and collecting localities are given in Table S1.

Outgroups belong to the Spirophorida since all previous *Demospongiae* ^p^ molecular phylogenetic studies place them in a strongly supported sister-order relationship with the Astrophorida [1], [4], [5], [21], [27] (see also the comprehensive COI, 18S and 28S phylogenetic *Demospongiae* ^p^ trees on the Sponge Genetree Server, www.spongegenetrees.org/, accessed on the 15^th^ of October 2010).

### Taxonomy

Specimens collected were identified to the genus and species level by P. Cárdenas, H. T. Rapp and J. R. Xavier. Identifications of specimens donated by other institutions were also checked. Astrophorida vouchers from previous studies [4], [24], [28], [29], [30] were re-examined by us or by others [31], [32] and in some cases, given new identifications (Table S2. Some of the voucher specimens sequenced have been morphologically described previously: *Pachymatisma* species [33] and all specimens collected in Panama [34]. The Norwegian Pachastrellidae specimens will be described and reviewed in a separate paper.

*Isops* and *Sidonops* are synonyms of *Geodia* [35]; *Isops* and *Sidonops* species of this study were therefore all transferred to *Geodia*. *Geodia neptuni* Sollas, 1886 has been synonymized with *Geodia vosmaeri* Sollas, 1886 [36]. *Erylus euastrum* has been transferred to the genus *Penares*, owing to molecular and morphological results [35]. The lithistid *Exsuperantia* sp. corresponds to *Racodiscula clava sensu* Topsent, 1892 from the Azores [37] which had been re-identified as *Rimella* sp. [38], later found to be a preoccupied genus [39]. Because *Thrombus abyssi* can have variable spicule morphologies [40], it is important to note that our specimens have amphiasters and trichotriaenes with an extension of the rhabdome.

### DNA extraction, amplification and sequencing

Two independent genes were used for this study: the Folmer fragment of the mitochondrial cytochrome c oxidase subunit 1 (COI) and the 5′ end terminal part of the nuclear 28S rRNA gene. These have previously been shown to give robust and congruent results for Geodiidae relationships [35]. DNA extraction from choanosome samples was performed using the Tissue Genomic DNA extraction kit (Viogene, Sunnyvale, CA, U.S.A.) in accordance with the manufacturer's instructions. A single centrifugation step was added just before pipeting the mixture into the columns in order to remove the spicules. For some species (*Pachastrella* sp. and *Stryphnus raratriaenus*), polymerase chain reactions (PCR) worked only when the DNA was extracted following a standard chloroform protocol extraction. The 5′ end region of COI (659 bp.) was amplified using LCO1490 and HCO2198 [41] (5 min/94°C; 5 cycles [30 s/94°C, 1 min30 s/45°C, 1 min/72°C]; 30–35 cycles [30 s/94°C, 1 min30 s/50°C, 1 min/72°C]; 7 min/72°C). C1′ASTR (5′–ACC CGC TGA ACT TAA GCA T–3′) [35] and the D2 (5′–TCC GTG TTT CAA GAC GGG–3′) [42] reverse universal primer were used to amplify a 768–832 bp. region of 28S comprising part of the C1 domain, and the total of the D1, C2 and D2 domains [5] (1 cycle [4 min/95°C, 2 min/59–60°C, 2 min/72°C]; 35 cycles [1 min/94°C, 45 s/59°C, 1 min/72°C]; 7 min/72°C). In some cases, C1′ASTR did not work and we used an intermediate primer instead: Ep1a' (5′–GGC AGA GGC GGR TGC ACC–3′) [5]. Sequences were then shorter, ca 690 pb (1 cycle [4 min/95°C, 2 min/59°C, 2 min/72°C]; 35 cycles [1 min/94°C, 45 s/59°C, 1 min/72°C]; 7 min/72°C). PCR products were purified using the ExoSAP-IT® kit (USB Europe, Staufen, Germany) or gel purified using a Gel-M™ Gel Extraction System (Viogene). Cycle sequencing was performed using a dye-labeled dideoxy terminator (Big Dye® Terminator v3.1, Applied Biosystems, Foster city, CA, U.S.A.). Products were analyzed using an ABI Prism 3700 DNA Analyzer (Applied Biosystems). The Astrophorida origin of the sequences was checked by BLAST searches (http://blast.ncbi.nlm.nih.gov).

### Sequence alignments and phylogenetic analyses

The COI data matrix includes 118 sequences (with outgroups) of which 86 are new. 245/660 characters are parsimony informative. The 28S data matrix includes 108 sequences of which 80 are new and 9 are lengthened since Cárdenas et al. [35]. 381/864 characters are parsimony informative. COI sequences were manually aligned in Se-Al v2.0a11 [43]. 28S sequences were first automatically aligned using MAFFT v.6.705 [44] with default parameters, implemented in SeaView v.4.1 [45]. Four insertion-deletion regions (4–20 bp long) in the D2 domain were ambiguous to align and regional realignments using the MAFFT's ENSI strategy were computed on these four regions. The alignment was subsequently improved visually using Se-Al.

Altogether, maximum likelihood (ML) analyses were conducted on four datasets: COI, COI amino-acids, 28S and 28S+COI. 28S (D1-D2) and COI have been shown to evolve at similar rates [35], so the two datasets were concatenated in a single matrix containing a total of 148 Astrophorida specimens (29 genera, 2 sub-genera, 89 species) and 1,527 characters, of which 811 are constant, 110 are parsimony uninformative and 606 parsimony informative. For some species we had both markers, but in different specimens from the same region (e.g. *Stelletta normani* from Western Norway, *Geodia megastrella* from the Hebrides Islands, *Pachastrella ovisternata* from the NEA). The sequences of these specimens were concatenated in the final matrix. Overall, we had a sequence for both genes for 67 specimens and 59 species of Astrophorida. ModelTest 3.7 [46] and ProtTest 2.4 [47] were used to find the most appropriate models of evolution respectively for the nucleotide datasets and the amino-acid dataset. For COI, COI amino-acids, 28S and COI+28S, the models were respectively (according the Akaike Information Criterion): HKY+I+G, metREV+G, TrN+I+G and GTR+I+G. For ML runs and bootstrap analyses we used GARLI v.0.96 [48] and Grid computing [49] through The Lattice Project [50], which includes clusters and desktops in one encompassing system [51]. A Grid service for GARLI was developed using a special programming library and associated tools [52]. Following the model of Cummings et al. [53], who used an earlier Grid computing system [54], the Astrophorida data matrix was distributed among hundreds of computers, where the analyses were then conducted asynchronously in parallel. 100 ML search replicates were run for each dataset. Each replicate was run with a random starting topology and for 5,000,000 generations. Lscores of the 100 best trees from each replicate were re-estimated in PAUP* 4.0b10 [55] and trees were compared using the Symmetric Difference (Robinson-Foulds) tree distance metric, essentially to make sure the best trees collected had similar topologies. 2,000 bootstrap replicates were conducted for each of these four datasets.

To investigate spicule evolution, we reconstructed the microscleres and megascleres states at ancestral nodes on the molecular tree using likelihood reconstruction methods under the Mk1 model [56], with the help of Mesquite 2.74 [57] and a morphological matrix with 13 characters combined from our observations and from species descriptions in the literature (Table S3).

Astrophorida species can be found at various depths. To investigate a possible relationship between depth, evolution of spicules and/or phylogeny, we have color-coded shallow and deep-water species (>100 m) in the character states reconstructions. Shallow submerged cave environments are prone to harbor deep-water sponge species [58], [59], so specimens collected in shallow Mediterranean caves were considered as deep-water species if records outside caves were in deep-water: this concerns *Penares euastrum*, *Calthropella pathologica*, *Discodermia polymorpha* and *Neophrissospongia nolitangere*. *Stelletta lactea* and *Penares helleri* were the only species to appear in both shallow and deep waters.

### Phylogenetic classification of the Astrophorida

Following our effort to revise sponge classification as we construct new molecular phylogenies [35], we followed the principles of phylogenetic nomenclature under the rules of the *PhyloCode v.4c* (http://www.ohiou.edu/PhyloCode/) to build a phylogenetic classification based on our results. Phylogenetic nomenclature provides the opportunity to propose taxonomical changes while waiting for independent evidence to confirm them, and before implementing those changes to the more widely used rank-based Linnaean classification. This is particularly important to reduce the phylogeny/classification gap. It is also very useful for intra-genera relationships (e.g. in *Geodia*) where the rank-based classifications are insufficient to name and describe all the clades present [35]. We named clades that have a bootstrap higher than 70 in the 28S+COI analysis. For the use and establishment of clade names, including species names, we will follow Cárdenas et al. [35].

## Results

The best tree resulting from the COI amino-acids analyses is poorly resolved with very few supported clades (Fig. S1). The best trees from the COI analyses (Fig. S2) and the 28S analyses (Fig. S3) are well resolved and congruent except for a few deep poorly-supported nodes. The main topology differences between the COI and 28S trees are: i) *Alectona* clusters with the Spirophorida outgroups (28S) or with the rest of the Astrophorida (COI); ii) *Thenea* and *Poecillastra*+*Vulcanella* form a monophyletic group (28S) or not (COI); iii) *Geodinae* ^p^ Sollas, 1888 [Cárdenas et al., 2010] cluster either with the *Erylinae* ^p^ Sollas, 1888 [Cárdenas et al., 2010] (COI) or with some Ancorinidae (28S).

**Figure 2:**
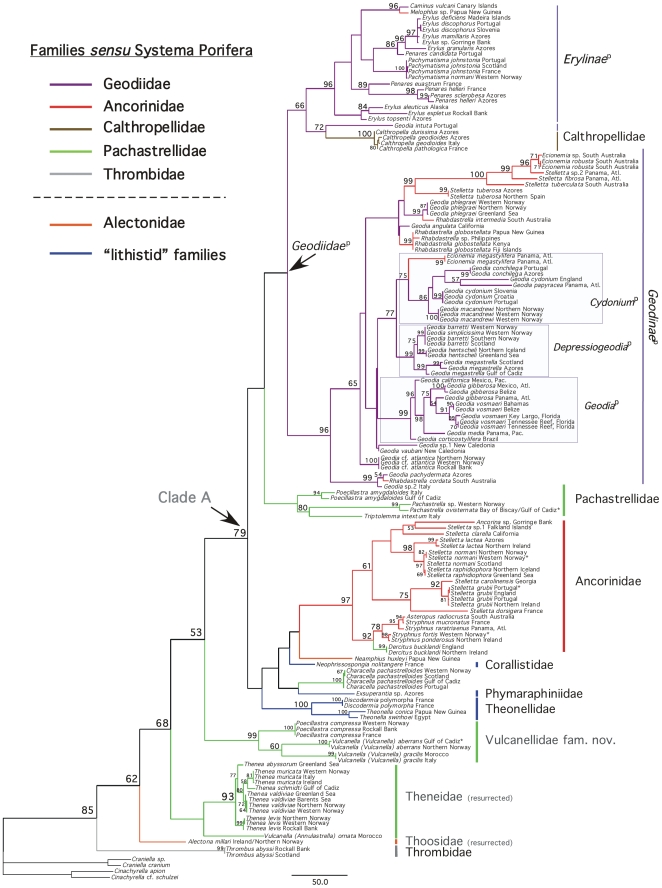
Maximum-likelihood phylogeny of the *Astrophorida* ^p^ using 28S+COI partial sequences from 153 taxa (89 species). Bootstrap nodal support values >50 are given at the nodes (2,000 replicates). Species names (according to the Linnaean classification) and sampling localities are given in Table S1. Names established under the *PhyloCode* are in italics and identified with the symbol ‘p’.

The best tree from the 28S+COI analyses (Fig. 2) is fairly close to the COI tree except for the poorly-supported positions of *Pachastrella*, *Poecillastra* and *Vulcanella* (*Vulcanella*). From now on, we will present the results of the best tree obtained with the 28S+COI dataset (Fig. 2), unless significant topology differences were observed in the analyses of the other datasets. Parameters estimated by GARLI for the best 28S+COI tree were (lnL = −19335.557146; A = 0.191611; C 0.247736; G = 0.290797; T = 0.269856; *R*-matrix = (1.137933 3.456486 1.476993 0.844493 4.787326); pinv = 0.367474; α = 0.557592). Out of the 100 best trees (each obtained from a different ML replicate), the first 66 trees (19335.56<PAUP* lnL<19336.10) had only minor topology differences, essentially within the *Geodinae* ^p^ and the *Erylinae* ^p^. The best tree presented and discussed here is the one with the highest score (−lnL = 19335.56); it is also representative of more than half of the trees found.

### Geodiidae, Calthropellidae and Ancorinidae

Astrophorida (including lithistids, *Alectona* and *Neamphius*) was monophyletic in all analyses except for the 28S analyses, were *Alectona* was within the Spirophorida outgroups. Out of the 100 best trees retrieved from the 28S+COI analyses, the first 76 trees suggested identical topologies concerning the relationships between the Geodiidae, Calthropellidae and Ancorinidae. The Geodiidae and the Ancorinidae were not monophyletic, while the Calthropellidae was monophyletic (but with only one genus sampled: *Calthropella*). Some Ancorinidae genera were distributed within the Geodiidae while the rest clustered in the Ancorinidae *sensu stricto*. Furthermore, some of the Ancorinidae genera appeared polyphyletic: i) within *Geodinae* ^p^ (*Ecionemia* and *Rhabdastrella*), or ii) distributed between *Geodinae* ^p^ and *Ancorinidae* ^p^ (*Stelletta*). *Melophlus* sp., another Ancorinidae, clustered with *Caminus vulcani* in the *Erylinae* ^p^.

*Geodiidae* ^p^ Gray, 1867 [Cárdenas et al., 2010] is poorly supported, but retrieved in the COI analyses (Fig. S2) and in the first 76 best trees of the 28S+COI analyses (Fig. 2). The 77^th^ best tree offers a new topology: ((*Geodinae* ^p^+Ancorinidae *s.s.*) *Erylinae* ^p^). When we go from tree 76 to tree 77 we go from lnL = −19937.93 to lnL = −19939.79, a significant jump in likelihood when compared with the lnL very slow decrease from tree 51 to tree 76. We therefore also ran constrained analyses on the 28S+COI dataset (100 ML replicates) forcing the *Geodinae* ^p^ and Ancorinidae *s.s.* together. The best constrained tree scored a lnL = −19339.79 (same as our tree number 77). An Approximately Unbiased (AU) test using CONSEL v.0.1j [60] showed that the best constrained and unconstrained trees were not significantly different (*P*-value = 0.395), so both topologies are plausible according to our molecular data. We should also note that the ((*Geodinae* ^p^+Ancorinidae *s.s.*) *Erylinae* ^p^) topology is also retrieved in the 28S analyses (Fig. S3). *Geodinae* ^p^ and *Erylinae* ^p^ were both strongly supported (bootstraps of 96). *Erylus* and *Penares* were both found polyphyletic, with most *Erylinae* ^p^ internodes poorly supported. Within *Geodinae* ^p^, *Depressiogeodia* ^p^ [Cárdenas et al., 2010] and *Geodia* ^p^ Lamarck, 1815 [Cárdenas et al., 2010] were strongly supported (boostraps of 99), while *Cydonium* ^p^ Fleming, 1828 [Cárdenas et al., 2010] was moderately supported (boostrap of 86). All species for which we had sampled more than one specimen were found monophyletic except for *Geodia cydonium* (the British specimens were clearly separated from the Mediterranean/Portuguese specimens, K2P distance = 0,04606), *Geodia gibberosa* (paraphyletic) and *Penares helleri* (paraphyletic). *Geodia simplicissima* and *Geodia barretti* had identical COI sequences.

A *Calthropella*+*Geodia intuta* clade appeared as sister-group to *Erylinae* ^p^. This topology was poorly supported (bootstraps of 66 and 72) but retrieved in all ML replicates.

### Ancorinidae *sensu stricto*

The Ancorinidae *s.s.* have the most recent common ancestors with lithistids, *Characella pachastrelloides* (Pachastrellidae) and *Neamphius huxleyi* (Alectonidae). The Ancorinidae *s.s.* included *Asteropus*, *Stryphnus*, *Ancorina* and some *Stelletta* (henceforth called *Stelletta sensu stricto*). *Stryphnus* and *Stelletta s.s.* appeared paraphyletic, the first one because of the placement of *Asteropus* sp., the second because of *Ancorina* sp.. *Dercitus bucklandi* (Pachastrellidae) was found basal to the *Stryphnus*+*Asteropus* clade. As detailed above, a few 28S+COI trees (with lower likelihoods) and the 28S analyses suggested that the Ancorinidae *s.s.* was sister-group to *Geodinae* ^p^.

### Pachastrellidae and lithistids

The Pachastrellidae appeared as a polyphyletic group distributed in four clades: clade 1) *Characella pachastrelloides*, clade 2) *Pachastrella*+*Poecillastra amygdaloides*+*Triptolemma intextum*, clade 3) *Poecillastra compressa*+*Vulcanella*(*Vulcanella*) and clade 4) *Thenea*/*Vulcanella*(*Annulastrella*). As a result, *Thenea* and *Pachastrella* were monophyletic while *Poecillastra* and *Vulcanella* were polyphyletic. *C. pachastrelloides* is grouping next to the lithistids. Clade 2 was found to be sister group to the *Geodiidae* ^p^ clade but this was very poorly supported (boostrap<50). Clade 2 moved closer to the *Erylinae* ^p^ and *Calthropella* in the COI and 28S analyses. Clade 3 and 4, both very well-supported, appeared closer to the base of the Astrophorida clade, but the nodes were moderately to poorly supported (bootstraps of 68 and 53). In the 28S analyses, Clade 3 and 4 form a poorly-supported monophyletic clade. In the COI analyses, Clade 3 is sister-group to the *Geodiidae* ^p^, the branch is very short and poorly-supported.

The lithistids were here limited to three families two of which (Corallistidae and Phymaraphiniidae) were only represented by a single species. *N. nolitangere* and *Exsuperantia* sp. were found close to *C. pachastrelloides* but this was poorly supported (bootstraps<50). With three species sampled, the Theonellidae was found monophyletic (bootstrap of 100).

### Thrombidae and Alectonidae

With two species sampled, the Alectonidae was found polyphyletic. *Alectona millari* branched between the Thrombidae and the rest of the Astrophorida. In the 28S analyses, *Alectona* was placed between the *Cinachyrella* and *Craniella* outgroups. *Neamphius huxleyi* was sister-group to the Ancorinidae *s.s.* but this association was not supported (bootstrap<50). In the COI analyses, *N. huxleyi* branched with the lithistids, but not far away from the Ancorinidae *s.s.*; this position was not supported either. *Thrombus abyssi* is the most basal Astrophorida, branching before *A. millari*.

### Maximum likelihood reconstruction of ancestral states

**Figure 3:**
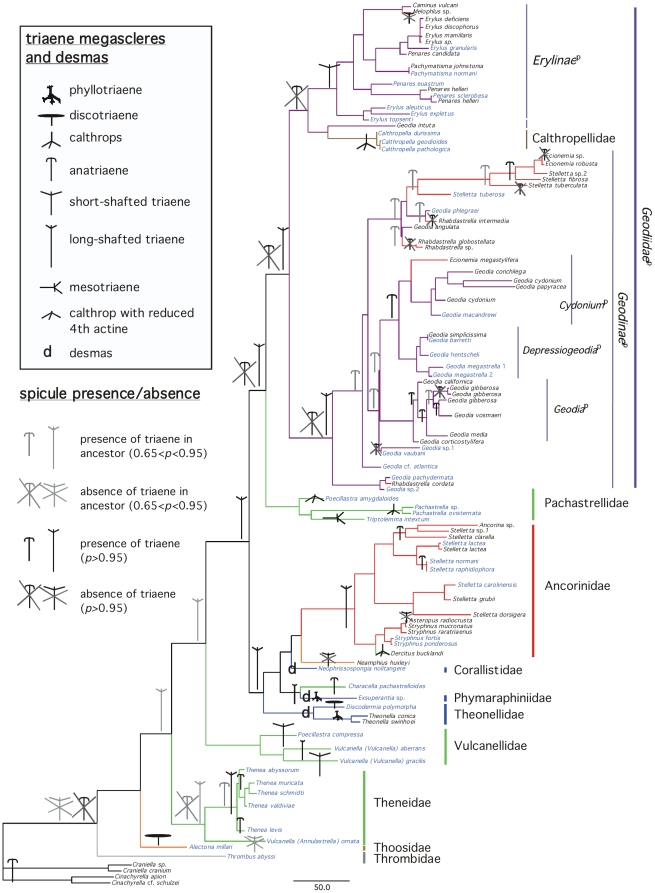
Presence and absence of megasclere spicules mapped on the *Astrophorida* ^p^ 28S+COI ML tree from **Figure 2**. The ML reconstructions of the ancestral conditions at the nodes were estimated using Mesquite 2.74. For the readers' convenience, species clades have been reduced to one sample (except in cases of para- or polyphyletic species). Species names in blue represent deep-water species. Species names in black represent shallow-water species. For the color-codes of the Astrophorida families *sensu Systema Porifera*, see Figure 2.

**Figure 4:**
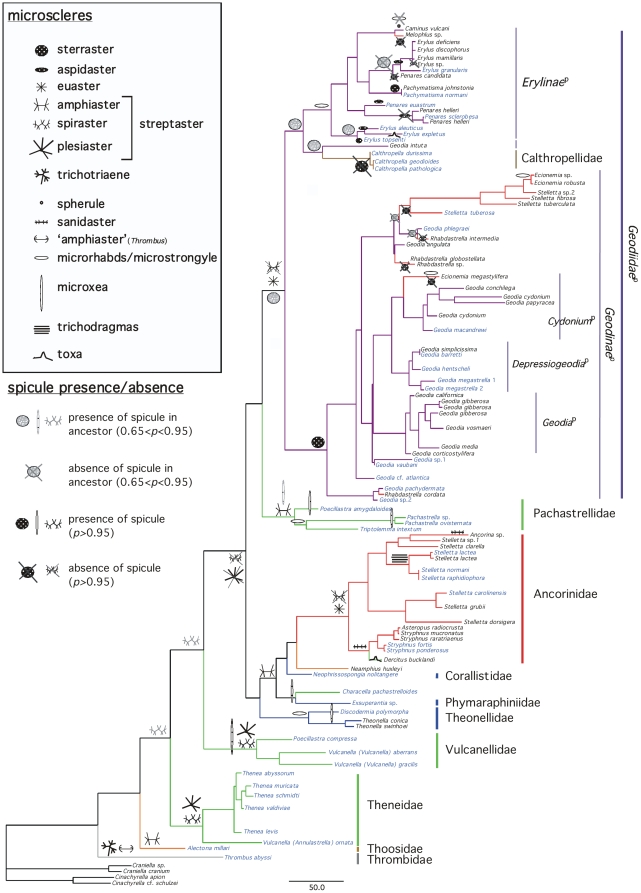
Presence and absence of microsclere spicules mapped on the *Astrophorida* ^p^ 28S+COI ML tree from **Figure 2**. The ML reconstructions of the ancestral conditions at the nodes were estimated using Mesquite 2.74. For the readers' convenience, species clades have been reduced to one sample (except in cases of para- or polyphyletic species). Species names in blue represent deep-water species. Species names in black represent shallow-water species. For the color-codes of the Astrophorida families *sensu Systema Porifera*, see Figure 2.

Mapping of the 13 characters on the molecular tree gave us 13 trees, each with relative probabilities for every character state for every node in the tree. We have summarized these results for megascleres (Fig. 3) and microscleres (Fig. 4) by only showing character states with 0.65>*p*>0.95, and *p*>0.95. Numerous cases of spicule convergent evolution and secondary losses are revealed.

On a total of 89 species sampled, we found 43 to be shallow and 46 to be deep-sea species. If we consider secondary losses of megascleres with *p*>0.95, we found 9 losses in shallow-species vs. 2 losses in deep-sea species (Fig. 3). We note there are no losses of triaenes in deep-sea species. If we consider secondary losses of microscleres with *p*>0.95, we found 14 losses in shallow-species vs. 5 losses in deep-sea species (Fig. 4).

Convergent evolution can be difficult to identify since we often have low probabilities for all character states in deep ancestors. With such an uncertain ancestor separating two clades, we cannot be sure that a spicule appearing in a clade is homologous to the same spicule type in the other clade, or not (e.g. microxeas, amphiasters). We nonetheless notice that convergent evolution is also quite frequent and concerns nearly all types of microscleres (amphiasters, toxas, sanidasters, euasters, aspidasters, microrhabds and possibly microxeas) and megascleres (short- and long-shafted triaenes, discotriaenes, phyllotriaenes, anatriaenes, calthrops). Desmas may have also appeared independently three times.

## Discussion

### Astrophorida and phylogenetic classification

**Figure 5:**
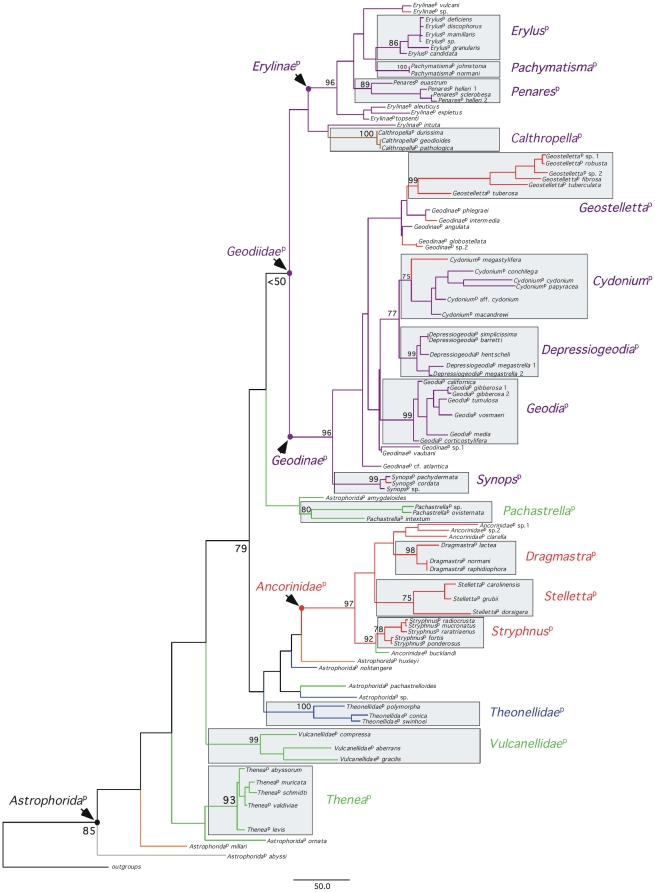
Phylogenetic classification of the *Astrophorida* ^p^ on the 28S+COI ML tree (cf. File S1 for definition of names). Species names are given according to the *PhyloCode* (Article 21.5). Bootstrap nodal support values of clades defined by the *PhyloCode* are given (2,000 replicates). For the color-codes of the Astrophorida families *sensu Systema Porifera*, see Figure 2.

A phylogenetic classification of the Astrophorida, henceforth named *Astrophorida* ^p^, is presented in File S1 and summarized in Figure 5. Names have been given to the well-supported clades (boostraps >70). Rank-based names have also been given to clades for which no names existed in the Linnaean classification. Moreover, new definitions of families and genera were also required. The revised Astrophorida Linnaean classification is presented in File S2.

Very early on, sponge taxonomists subdivided the Astrophorida between those that possessed streptaster and those that possessed euasters [15]: Streptosclerophorida and Euastrophorida respectively. Chombard et al. [5] previously found the Euastrophorida monophyletic and the Streptosclerophorida paraphyletic because they had mainly sampled *Geodiidae* ^p^ species, except for *Stryphnus mucronatus* that they had classified as a Streptosclerophorida (on the basis that its sanidasters were homologous to streptasters). However, our study suggests that both sub-orders are polyphyletic (irrespective of the nature of the sanidasters of *Stryphnus*). Therefore, we propose to formally abandon the two suborders Euastrophorida and Streptosclerophorida.

### Geodiidae^p^ and reallocated Ancorinidae

Since the last molecular phylogeny of *Geodiidae* ^p^ [35], we lengthened the 28S sequences and increased the sampling from 24 to 38 Geodiidae species and from 24 to 62 Geodiidae specimens. We also added species from phylogenetically close families (Ancorinidae and Calthropellidae). Clearly, *Geodiidae* ^p^ is poorly supported in our 28S+COI best tree (Fig. 2), but morphological data [35] and a majority of our 28S+COI best trees support the *Erylinae* ^p^+*Geodinae* ^p^ grouping. This is therefore the topology we will discuss in this paper. However, as we stated earlier (cf. Results), the alternative topology *Erylinae* ^p^(*Geodinae* ^p^+Ancorinidae) found in a few 28S+COI searches and 28S analyses could not be rejected on statistical grounds. The contentious *Geodiidae* ^p^ node should therefore be investigated further with additional molecular markers.

The Geodiidae is here redefined: it appears as a much larger family than expected since it includes genera from the Calthropellidae and Ancorinidae. This is surprising for a group whose monophyly and morphological synapomorphies appear quite clearly [35]. To understand this, we must consider the morphology of the unexpected groups. The Ancorinidae is partly composed of species which have the same set of spicules as the Geodiidae except for the presence of sterrasters (ball-shaped euasters, Fig. 1e). Consequently, these Ancorinidae may have never had sterrasters or they may have secondarily lost them. In the second case, these species should be reallocated within the Geodiidae.

*Penares* is one of these former Ancorinidae genera reallocated to the Geodiidae based on morphological, molecular and biochemical data [5], [35]. To understand this reallocation, it was hypothesized that *Penares helleri* had secondarily lost its sterrasters [5]. Our study confirms this reallocation by adding two other species of *Penares*. Furthermore, the latter double the frequency of the secondary loss of sterrasters since our results suggest that *Penares* is polyphyletic, just like its counterpart *Erylus*. Secondary loss of sterrasters therefore happened at least twice in two different newly named clades: *Penares* ^p^ (*P.* ^p^ *euastrum*, *P.* ^p^ *helleri* and *P.* ^p^ *sclerobesa*) and *Erylus* ^p^ (*E.* ^p^ *discophorus*, *E.* ^p^ *mamillaris*, *E.* ^p^ *deficiens*, *E.* ^p^ sp., *E.* ^p^ *granularis* and *E.* ^p^ *candidata*) (Fig. 4, Fig. 5). If it happened twice, it could have happened more, and this is what the placement of *Erylus* ^p^ sp. (an *Erylus* with no aspidasters) and other genera of Ancorinidae within the *Geodiidae* ^p^ suggest: *Melophlus* sp., *Rhabdastrella*, *Ecionemia*, and some *Stelletta* would have also lost their sterrasters (Fig. 4). As in the example of *Penares* ^p^, this is fairly easy to conceive since these Ancorinidae species share i) spicule repertoires identical to the *Geodiidae* ^p^ except for the presence of sterrasters, and often ii) a similar external morphology (e.g. oscule organization). Despite these similarities, the placement of the polyphyletic *Rhabdastrella* and *Ecionemia* within the *Geodinae* ^p^ is not straightforward.

Based on the possession of microrhabds in the cortex, Chombard et al. [5] wondered if *Ecionemia* should be reallocated to the *Erylinae* ^p^. Our analysis suggests that the three *Ecionemia* species sampled belong to the *Geodinae* ^p^, and are distributed in two groups. The two Australian *Ecionemia* group with some *Stelletta* — thus forming the new clade *Geostelletta* ^p^ — while *Ecionemia megastylifera* from the Caribbean is branching at the base of *Cydonium* ^p^. These three species of *Ecionemia* all share large spiny microrhabds in the cortex along with euasters. Since microrhabds are absent from all the other *Geodinae* ^p^ of this study, the origin of these microrhabds is uncertain at this point and may represent yet another case of morphological spicule convergence in sponges (Fig. 4). Other species of *Ecionemia*, with small sanidaster-like microrhabds (e.g. *E. acervus*, type species of the genus, *E. demera*, *E. walkeri*), might instead be linked to sanidaster-bearing *Ancorinidae* ^p^ as previously suggested [61], [62], [63]. In our opinion, the genus *Ecionemia* should therefore be kept valid for the remaining species of *Ecionemia* whose phylogenetic positions remain to be tested.

Based on its spicules and skeleton organization, *Rhabdastrella* has previously been suspected to be close to the Geodiidae [64] or even part of the Geodiidae [65]. Biochemical data also concurs with this result: isomalabaricane triterpenes have been found in *R. globostelletta* and *Geodia japonica* [66], [67]. *Rhabdastrella* species from our study are distributed in three groups: 1) *R. globostelletta* and *Rhabdastrella* sp. form a clade of uncertain position within the *Geodinae* ^p^, 2) *R. cordata* from Australia forms a strongly supported group with *Geodia* ^p^ *pachydermata* and *Geodia* ^p^ sp. 2, both from the Atlantic/Mediterranean area, and 3) *R. intermedia* forms a strongly supported clade with *Geodia* ^p^ *phlegraei*. *Rhabdastrella* species are characterized by sterrospherasters in the cortex. Sterrospherasters is a general ambiguous term that includes two main types of large euasters: i) very large spherasters with smooth conical rays, filling the whole cortex (e.g. *R. globostelletta* and *Rhabdastrella* sp.) or ii) sterrasters, sometimes with incompletely fused actines (e.g. *R. rowi*, *R. aurora*, *R. cordata*), placed in the endocortex. These morphological observations coupled with our results suggest that these sterrospherasters might actually be, in the first case, true spherasters — they resemble the ones found in the phylogenetically close *G.* ^p^ *phlegraei* and *G.* ^p^ *angulata* — and are, in the second case, true sterrasters. *Rhabdastrella* with true spherasters may therefore have secondarily lost their sterrasters (and these have been replaced by the large spherasters). In light of these results we expect all *Rhabdastrella* species to be redistributed in *Geodinae* ^p^. The genus *Rhabdastrella* is therefore not valid and should be synonymized with *Geodia*. As a consequence of the polyphyly of *Rhabdastrella*, the confusing spicule term ‘sterrospheraster’ should be once and for all rejected, as suggested before [68].

We should not be surprised to find Ancorinidae species with microrhabds such as *Melophlus* sp. grouping with *Caminus vulcani* (an *Erylinae* ^p^ with spherules) since it has been argued that spherules may have evolved from microrhabds [35]. Furthermore, like the rest of the *Erylinae* ^p^, *Melophlus* sp. has no ana/protriaenes. The phylogenetic position of *Melophlus* sp. among the *Erylinae* ^p^ may be further supported by biochemical data: sarasinoside M, a triterpenoidal saponin isolated from *Melophlus sarassinorum*, has strong similarities with the framework of Eryloside L, isolated in *Erylus lendenfeldi* [69].

To conclude, the reallocation of numerous Ancorinidae species in the Geodiidae calls for new definitions for these families (File S2).

### *Geodinae* ^p^

Most of the clades found in this study are identical to those found previously with fewer species and a shorter 28S fragment [35]. *Geodia* ^p^, *Cydonium* ^p^ and *Depressiogeodia* ^p^ were still strongly supported groups. The *Depressiogeodia* ^p^+*Cydonium* ^p^ clade, poorly supported in Cárdenas et al. [35], was better supported here (bootstrap of 77), it exclusively grouped Atlantic species. In the following paragraphs, we will go through these clades and discuss new taxonomical results that have arisen due to the addition of new species since Cárdenas et al. [35].

The addition of *Geodia* ^p^ *corticostylifera* from Brazil confirmed that the *Geodia* ^p^ include species from North and South America, from the Atlantic and Pacific sides. Different clades of *Geodia* ^p^ *vosmaeri* (former *G.^p^ neptuni*) appeared, two from Florida, another from Belize+Bahamas suggesting i) a strong geographical structure and that ii) the molecular markers used may be suited for future intra-specific studies. Our results confirmed that *Geodia* ^p^ *gibberosa* represented a species complex, as previously hypothesized with morphological observations [34]. We propose that *G.* ^p^ *tumulosa* Bowerbank, 1872 (a synonym of *G.* ^p^ *gibberosa*) should be resurrected for the mangrove specimen from Panama. Its tumulose shape is clearly different from the barrel-shape of our reef specimens from Belize and Mexico, more similar to the shape of the holotype of *G.* ^p^ *gibberosa* (specimen MNHN DT-608).

*Geodia* ^p^ *conchilega* and *E. megastylifera* are part of *Cydonium* ^p^ so this clade still gathers Atlanto-Mediterranean species. The polyphyly of *Geodia* ^p^ *cydonium* calls for a revision of this species whose taxonomical history is old and complex.

*Geodia* ^p^ *megastrella* is part of the *Depressiogeodia* ^p^. This clade thus remained a Northeast Atlantic deep-water species group. The inclusion of *G.* ^p^ *megastrella* in the *Depressiogeodia* ^p^ also confirmed a suggested morphological synapomorphy of the group: a deep preoscule lacking sterrasters in its cortex [35]. It should be noted that the *G.* ^p^ *megastrella* ZMBN 85208 (Scotland) and ZMAPOR 21654 (Azores) both had a distinct large deletion (35 bp long) in their 28S D2 domain while ZMAPOR 21231 (Morocco) appeared to have a slightly different sequence, notably without the deletion. This specimen's morphology needs to be further investigated as *G.* ^p^ *megastrella* may represent a species complex.

The two deep-water *Geodia* ^p^ species from New Caledonia grouped together but this is poorly supported. The most basal *Geodinae* ^p^ was a strongly supported clade named *Synops* ^p^ grouping *G.* ^p^ *pachydermata*, *Geodia* ^p^ sp. 2 and *R. cordata*. The surprising phylogenetic position of *Geodia intuta* with *Calthropella* will be discussed below. The positions of other *Geodinae* ^p^ species (e.g. *G.* ^p^ *phlegraei*, *G.* ^p^ *angulata*) were poorly supported and uncertain (different positions in different trees) so we cannot discuss their taxonomy at this point.

### *Erylinae* ^p^

*Erylinae* ^p^ was a very strongly supported group (boostrap of 96). The monophyly of *Erylus* has been previously challenged by morphological and molecular data [33], [35]. Our results suggested that it was a polyphyletic genus, mixed with *Penares*, *Caminus*, *Melophlus* and *Pachymatisma* ^p^ species. *Erylus* species were distributed in three clades: *Erylus* ^p^ (‘*nomen cladi conversum*’ because it holds the type species of *Erylus*: *E.* ^p^ *mamillaris*), *Penares* ^p^ (‘*nomen cladi conversum*’ because it holds the species type of *Penares*: *P.* ^p^ *helleri*) and *Erylus*1 (temporary name for the clade including *E. aleuticus*+*E. expletus+E. topsenti*, poorly supported). If *Erylus* is polyphyletic, the most parsimonious scenario is that flattened sterrasters ( = aspidasters) have appeared independently at least three times; this is also suggested by our character reconstruction using ML methods (Fig. 4). Our study has not revealed the identity of *Erylus* ^p^ sp. collected in the Gorringe Bank [25]. *Erylus* ^p^ sp. which has lost its aspidasters was part of the *E.* ^p^ *mamillaris*/*discophorus* complex, but more rapidly evolving markers are required to fully understand this group.

### Calthropellidae and *Geodia intuta*

The association of calthrops and euasters essentially characterizes the Calthropellidae. According to some morphologists, the Calthropellidae do not really have characters of their own and should be within the Ancorinidae [70], [71], [72]. However, the first molecular evidence suggested a sister-group relationship between the Calthropellidae and the Erylinae [5]. Although the *Erylinae* ^p^(*G. intuta*+*Calthropella* ^p^) association was weakly supported (bootstrap of 66) it was present in all our trees obtained from the 100 ML searches. Furthermore, the external morphology of *Calthropella* ^p^ *geodioides* and some basal *Erylinae* ^p^ species (e.g. *E. expletus*) is quite similar: they are massive sub-spherical sponges with numerous white uniporal oscules on the top surface. We propose to reallocate the Calthropellidae to the Geodiidae by downgrading them to a sub-family: the Calthropellinae. *Paxataxa* and *Corticellopsis* are the other genera of the Calthropellinae since *Chelotropella* has been reallocated to the Ancorinidae [73]. Sequences of *Pachataxa* and *Corticellopsis* are therefore needed to confirm the monophyly and the position of this group.

The clustering of *Geodia intuta* with *Calthropella* ^p^ was surprising, but less so when reconsidering its external and spicule morphologies. Like *Erylus* ^p^ and *Penares* ^p^, *G. intuta* is a massive sub-hemispherical sponge with a smooth cortex, it is easily compressible, and has a rather confused skeleton organization. It was originally described as an *Isops* because of its uniporal oscule and pores. According to our observations, the oscule actually leads to a branching atrium, similar to the ones found in *Erylus* ^p^, *Penares* ^p^ or *Caminus*. This prompted von Lendenfeld [74] to describe it in a new genus, as *Caminella loricata*, before it was synonymized with *Geodia intuta* [75]. Moreover, it has long-shafted triaenes (as in the *Geodinae* ^p^) but no ana/pro/mesotriaenes (as in the *Erylinae* ^p^). It has spherasters in the ectocortex and globular sterrasters in the endocortex. Globular sterrasters are also present in many *Erylinae* ^p^ (e.g. *Caminus*, *Pachymatisma* ^p^, *E. topsenti*). As for spherasters, they resemble the spherules found in *C. vulcani* (an *Erylinae* ^p^) or *Calthropella* ^p^ *durissima*. All in all, although *G. intuta* shares many characters with some *Erylinae* ^p^ (*Erylus* ^p^, *Penares* ^p^, *Caminus*), the presence of long-shafted triaenes and the absence of microrhabds suggest that it is not an *Erylinae* ^p^. Therefore, we decided to resurrect the Geodiidae genus *Caminella* von Lendenfeld, 1894 to welcome this species. On the other hand, we will wait for further data to confirm its phylogenetic position and name the *G. intuta*+*Calthropella* ^p^ clade.

### *Ancorinidae* ^p^

Ancorinidae *sensu stricto* form a well-supported clade henceforth named *Ancorinidae* ^p^. *Stelletta* species were distributed in three *Ancorinidae* ^p^ clades: clade 1) (*Ancorina* sp.+*Stelletta* sp. 1)+*Stelletta clarella*, clade 2) (*Stelletta normani*+*Stelletta raphidiophora*)+*Stelletta lactea* and clade 3) (*Stelletta grubii*+*Stelletta carolinensis*)+*Stelletta dorsigera*. Clade 1 was poorly supported (bootstrap<50). Clade 2 clustered three Northeast Atlantic species; it was very well supported by our data (bootstrap of 98) and by the synapomorphy of trichodragmas (raphides in bundles) (Fig. 4): it was therefore named *Dragmastra* ^p^. Clade 3 held the type species of the genus (*S. grubii*) so it was named *Stelletta* ^p^. It should be noted that *S. dorsigera* does not group with *S. grubii* in the 28S analyses (Fig. S3). The unstable position of *S. dorsigera* may be due to the fact that the *Stelletta* COI sampling is quite poor with respect to the *Stelletta* 28S sampling. The grouping of clade 1+*Dragmastra* ^p^ is poorly supported or absent (28S analyses) but we nonetheless note that all of these species have dichotriaenes, except for *Ancorina* sp.. Conversely, species in the *Stelletta* ^p^ clade do not possess dichotriaenes. Instead, 28S analyses fully support a *Dragmastra* ^p^+*Stelletta* ^p^ clade (Fig. S3).

Since *Ancorina* and *Stryphnus* share similar spicule repertoires [34], notably the presence of sanidasters (Fig. 4), we were expecting them phylogenetically closer to each other than here observed. But the grouping of *Ancorina* sp. with two *Stelletta* species was poorly supported and may be due to the poor sampling of these speciose genera.

The close relationship between *Asteropus* and *Stryphnus* has often been discussed [15], [34], [76], [77], [78], [79]. Both genera have similar spicules, except for triaenes that *Asteropus* would have secondarily lost (Fig. 3). For the first time, the synonymy of *Asteropus* with *Stryphnus* is confirmed by molecular results. Therefore, we formally propose that *Asteropus* becomes a junior synonym of *Stryphnus* and name this clade *Stryphnus* ^p^.

The presence of *Dercitus bucklandi* — a Pachastrellidae with calthrops, sanidasters and toxas — within the *Ancorinidae* ^p^ is once more supported by morphological data. *Dercitus* (*Stoeba* included) and *Stryphnus* notably share sanidasters, large spherulous cells, and a similar aquiferous system [70], [75], [80]. But other authors had considered that the origin of the toxas being ambiguous, emphasis should instead be placed on the presence of calthrops, which had brought *Dercitus* closer to the Pachastrellidae [15], [81], [82], [83]. *D. bucklandi* as an *Ancorinidae* ^p^ suggests that toxas would have originated from asters, as previously hypothesized [75]. The modification of oxyasters into toxa-like spicules is actually quite common in the *Astrophorida* ^p^ (e.g. *Erylus nummulifer*, *Erylus expletus*, *Geodia apiarium*, *Erylus papulifer*, *Rhabdastrella oxytoxa* and *Stelletta toxiastra*). The difference between the latter and *D. bucklandi*, which troubled morphologists, is that toxas in *D. bucklandi* have completely lost trace of the original euaster centrum. The position of *D. bucklandi* also shows that its sanidasters are homologous to those of *Stryphnus* ^p^ (Fig. 4). Unfortunately, we did not get 28S sequences for *D. bucklandi* and the strongly supported *Stryphnus* ^p^+*D. bucklandi* clade needs to be confirmed before resurrecting the Sanidasterinae Sollas, 1888, characterized by the possession of sanidasters. Furthermore, *Stoeba* (not sampled here) having been synonymized with *Dercitus* [73], we can be confident that *Stoeba* species should also be reallocated to the *Ancorinidae* ^p^.

### The polyphyletic Alectonidae

The Alectonidae Rosell, 1996 (Hadromerida) are excavating sponges recently separated from the rest of the Clionaidae d'Orbigny, 1851 notably due to the possession of amphiasters or microrhabds, and absence of tylostyles. *Alectona* are known to produce a unique type of larva in the Porifera: an armored planktonic larva ( = hoplitomella larva) with discotriaenes [17], [18]. These are then lost by the adult, which settles and bores into biogenic substrata such as calcareous rocks or coral. The association of triaenes and amphiasters suggest that *Alectona* should be placed near or within the *Tetractinellida* ^p^ [Borchiellini et al., 2004] [17], [84]. A 28S (D1-C2) phylogenetic study then showed that the Alectonidae *sensu* Rützler [19] is polyphyletic and that *Alectona millari* belonged to the *Tetractinellida* ^p^ [16]. Our data not only confirmed this but also suggested that the Alectonidae genera *Alectona* and *Neamphius* belonged to the *Astrophorida* ^p^. In the 28S+COI analyses, *A. millari* branched after *Thrombus abyssi*, an acknowledged *Astrophorida* ^p^. In the 28S analyses, *Alectona* appeared within the Spirophorida outgroups branching between *Cinachyrella* and *Craniella* (Fig. S3), but the node between *A. millari* and *Craniella* sp. is not supported, and the branch is short. This result may be due to the fact that the *Alectona* 28S sequence is significantly shorter (409 bp.: D1-C2 domains) than the others sequences from this study. The ambiguous position of *Alectona* certainly deserves further investigation as it may represent a pivotal evolutionary step between Astrophorida and Spirophorida.

Having amphiasters but no triaenes, *Neamphius huxleyi* (the single species of its genus) has also been suspected to be an Astrophorida by morphologists [15]. According to our results it may be close to *Characella* and the lithistids. This is further supported by biochemical data showing that *N. huxleyi* and Astrophorida lithistids (*Callipelta* sp., *Theonella mirabilis* and *Theonella swinhoei*) share cyclic peptides and depsipeptides with cytotoxic and antiviral effects, notably with HIV-inhibitory activity [85], [86]. However, the position of *N. huxleyi* being equivocal and poorly supported, we propose to temporarily consider it as *incertae sedis*.

Our results also have consequences for the rest of the Alectonidae genera. Following Borchiellini et al. [16], we advocate the reallocation of *Thoosa* along with *Alectona*. *Delectona* might also join them since it shares amphiasters and toxas with *Thoosa*. These three genera (representing ca 29 species) would group in the Thoosidae Rosell and Uriz, 1997, here resurrected. The position of the rest of the Alectonidae (*Spiroxya*, *Dotona* and *Scolopes*) is at the moment uncertain although *Spiroxya* and *Dotona* are suspected to be phylogenetically close to each other [19]. On the Sponge Gene Tree Server (www.spongegenetrees.org [87], accessed on the 15th of October 2010), a phylogenetic 28S (B9-B21) tree of the Demospongiae suggested that *Spiroxya levispira* should remain close to the Placospongidae and the Trachycladidae (Hadromerida).

### Thrombidae

Since Lévi [82], the puzzling Thrombidae have been linked to the Astrophorida, based on their unique amphiasters and trichotriaenes. With the discovery of *Yucatania sphaerocladoides*, it appeared clear that *Thrombus* species had secondarily lost their triaenes [88], which confirmed that they belonged to the *Tetractinellida* ^p^. Our study showed that *Thrombus abyssi* is alone, at the base of the *Astrophorida* ^p^ tree which suggests, as for *Alectona*, the key role of this group in understanding how and when the *Astrophorida* ^p^ originated.

### The Pachastrellidae and the lithistids

The latest revision of the Pachastrellidae includes 12 genera [81] which share streptasters (rays proceeding from an axis that can be straight or spiral, Fig. 1e) and do not have euasters (rays radiating from a central point, Fig. 1e). Topsent [80] suggested that the Pachastrellidae could be subdivided between those that share a diverse set of streptasters (*Thenea*, *Vulcanella*, *Poecillastra*, some Corallistidae) and those whose streptasters are mainly restricted to amphiasters (rays radiating from both ends of a straight shaft, Fig. 1e) (*Pachastrella*, *Characella*, most Astrophorida lithistids). However, in our study, none of these groups were monophyletic (Fig. 2). We sampled six Pachastrellidae genera and they were distributed in five different clades: clade 1) *Dercitus* was reallocated to the *Ancorinidae* ^p^ (cf. above); clade 2) *Characella* appeared at the base of the *Ancorinidae* ^p^ along with lithistids and *Neamphius*; clade 3) *Poecillastra amygdaloides+Pachastrella+Triptolemma* was the sister clade of the *Geodiidae* ^p^. Although the positions of *Characella* and clade 3 were poorly supported and unstable depending on the dataset (Fig. S2, S3), they were clearly separated from the other Pachastrellidae genera branching further down in the tree: clade 4) *Poecillastra*+*Vulcanella*(*Vucanella*) and clade 5) *Thenea*+*Vulcanella*(*Annulastrella*). Clearly the Pachastrellidae were built on a plesiomorphy (the streptasters) and the family must be revised.

*Characella* is defined by amphiasters and at least two categories of monaxonic spicules (microxeas, microstyles, microstrongyloxeas) while *Poecillastra* is defined by a diverse set of streptasters (spirasters, metasters and plesiasters) and microxeas in a single category [81]. As *Characella* has been occasionally difficult to characterize with respect to *Poecillastra*, morphologists have questioned their validity [72], . Their definitions may overlap and many species are found to be “intermediate”, with characters of both genera (e.g. *Poecillastra saxicola*). According to our results, *Characella* was clearly separated from *Poecillastra* and phylogenetically closer to amphiaster-bearing lithistids. The definitions of *Characella* and *Poecillastra* should therefore prioritize the nature of streptasters and consider the number of categories of microxeas as a less reliable character, since these can be more ambiguous to characterize (cf. new definitions in File S2). Due to a lack of robustness, we propose to have *Characella* as *incertae sedis* at the moment, although we suspect that it could be allocated to a lithistid family in the future.

According to the *ICZN* and our results, the Pachastrellidae name should be kept for the *Pachastrella*+*Triptolemma* clade, henceforth named *Pachastrella* ^p^. Until further molecular data, we propose to include *Poecillastra amygdaloides* in this newly defined Pachastrellidae (File S2), although its position was poorly supported. *P. amygdaloides* has calthrops: this species and its synonym *Poecillastra debilis* had therefore originally been described as *Pachastrella* [90]. But *P. amygdaloides* was moved to *Poecillastra* because of its atypical triactinal calthrops, with a reduced fourth actine, later considered to be a modified triaene [15], [80]. Its sister-group position with *Pachastrella* ^p^ is supported by its spicule characters which seem intermediate between the *Poecillastra*+*Vulcanella*(*Vulcanella*) clade and *Pachastrella* ^p^: i) plesiasters (most of them are amphiaster-like) and ii) no microstrongyles. Other species (not sampled here) share the triactinal calthrops with *P. amygdaloides*: *Poecillastra nana*, *Poecillastra connectens* and *Characella capitolii*. We propose to resurrect *Nethea* Sollas, 1888 (originally defined as resembling *Poecillastra* but with triaenes with an underdeveloped rhabdome) to welcome these species. *Triptolemma* are cryptic excavating species penetrating the tissue of other sponges or coral. Many morphological characters support the *Pachastrella* ^p^ clade claimed by Topsent [80]. *Triptolemma* are characterized by short-shafted mesotriaenes of all sizes, which can be also produced by some *Pachastrella* species (e.g. *P. ovisternata*). Microscleres of *Triptolemma* are streptasters (from only amphiasters to a diverse set), microstrongyles and even microrhabdose streptasters [91]. These last two microscleres are apomorphies shared with *Pachastrella*. *Brachiaster* (not sampled here) surely belongs to this clade since it also produces short-shafted mesotriaenes, microstrongyles and amphiasters [92].

*Thenea*, *Vulcanella*, and *Poecillastra* share a diverse set of streptasters [80]. *Poecillastra*+*Vulcanella*(*Vulcanella*) further share i) an oscule area surrounded by cloacal oxeas (in *Poecillastra compressa* this area has expanded over a whole side of the sponge but the cloacal oxeas are still there), ii) an abundance of spiny microxeas, iii) a reduction of the triaenes to short-shafted triaenes or calthrops (even if long-shafted triaene species also exist) and iv) an absence of pro/anatriaenes (except in *Poecillastra rudiastra*). In order to welcome this very well supported clade named *Vulcanellidae* ^p^, we created the Vulcanellidae fam. nov. (File S2). On the other hand, the *Thenea*+*Vulcanella*(*Annulastrella*) clade was poorly supported (bootstrap<50). And yet, these two genera share i) large plesiasters and ii) absence of microxeas. For the time being, the Theneidae Carter, 1883 is resurrected to welcome these two genera. Also, *Vulcanella*(*Annulastrella*) needs to be upgraded to genus since it was clearly separated from *Vulcanella*(*Vulcanella*). The *Thenea* clade, here named *Thenea* ^p^, is very well supported (boostrap of 93) and also one of the few clades supported by the COI amino acid analyses (tree not shown). It groups species that share i) a characteristic external morphology (massive, hispid mushroom shape, Fig. 1d), with ii) a typical poral area, iii) long-shafted dichotriaenes (never calthrops), iv) an abundance of pro/anatriaenes and v) a system of roots to grow on muddy bottoms. Based on morphology, *Cladothenea* (not sampled here) should belong to this clade [81]. The Theneidae and the Vulcanellidae fam. nov. may i) form a poorly-supported clade (28S analyses, Fig. S3), ii) have a paraphyletic relationship (28S+COI tree, Fig. 2) or iii) be further apart (COI analyses, Fig. S2). All of these poorly supported topologies emphasize that relationships between these two families remain to be investigated.

As previously suggested by morphological [10], [15], [70], [93], [94], [95] and molecular data [5], [12], [13], our phylogeny confirmed that some lithistids belong to the *Astrophorida* ^p^. The *Discodermia*+*Theonella* clade named *Theonellidae* ^p^ was strongly supported (bootstrap of 100). According to morphology and a previous 18S phylogenetic study, *Racodiscula* may also be part of the *Theonellidae* ^p^ [13]. We note that *Discodermia* has microxeas and microrhabds while *Characella* (phylogenetically close to *Discodermia* in our tree) has two sizes of microxeas. The microrhabds of the *Theonellidae* ^p^ might therefore be homologous to the small microxeas of *Characella*. We also notice that the microrhabds of *Discodermia* are similar to the ones found in *Pachastrella* (e.g. *Discodermia proliferans*): these might also be homologous. *Exsuperantia* sp. (Phymaraphiniidae) is morphologically very close to the *Theonellidae* ^p^, but it has trider desmas instead of tetraclone desmas. *Exsuperantia* sp. either groups with *Characella* (28S+COI and 28S dataset), or with *N. huxleyi* (COI analyses). In both cases, the support was low. Morphological [82] and molecular [13] data suggest that the Corallistidae is a sister-group to the Theonellidae. Because of the low supported nodes between our lithistids this cannot be excluded: the position of *Neophrissospongia nolitangere* (Corallistidae) is unsure but certainly close to the other lithistids. Our results also hint that desmas have appeared independently in different Astrophorida lithistid groups (at least four times, if we would consider *Brachiaster*, not sampled here) (Fig. 3). This would not come as a surprise since desmas have appeared independently in other sponge orders as well [96]. It should be emphasized that, in our opinion, 8 out of the 13 extant lithistid families are of Astrophorida affinities (Corallistidae, Isoraphiniidae, Macandrewiidae, Neopeltidae, Phymaraphiniidae, Phymatellidae, Pleromidae, Theonellidae) representing ca 128 species [6]. A majority of them possess amphiaster streptasters while the remaining groups have additional spirasters (Corallistidae, *Pleroma*) or no asters (Macandrewiidae, *Discodermia*, *Theonella*). Therefore, although *Astrophorida* ^p^ lithistids do not seem to form a natural group, we can be certain that they all radiated along with amphiaster-bearing *Astrophorida* ^p^ (*Characella*, *Pachastrella*, *Triptolemma*, *Brachiaster*, and *Neamphius*). If they have a closest common ancestor with the *Ancorinidae* ^p^, the *Geodiidae* ^p^, or both, is still unclear at this point.

The node following that of the Vulcanellidae may be of importance since it supports, albeit moderately, a clade comprising amphiaster- and euaster-bearing *Astrophorida* ^p^ (Fig. 4), temporarily called ‘clade A’ (Fig. 2). Our study thus reveals for the first time the importance of amphiasters in *Astrophorida* ^p^ aster evolution, as an intermediate step between spirasters and euasters. The shortening of the amphiaster central shaft may represent an essential and preliminary stage to the appearance of euasters. Clade A includes all the *Astrophorida* ^p^ except for the Vulcanellidae, the Theneidae, *Alectona* and *Thrombus*, but since the position of the Vulcanellidae is unstable, so is the content of clade A. We thus refrain from formally naming clade A and wait for confirmation from other molecular markers. *Lamellomorpha strongylata* Bergquist, 1968 *incertae sedis* (not sampled) lacks triaenes and possesses only two types of microscleres: spiny microstrongyles and amphiaster-like streptasters. This species could therefore belong to the amphiaster/euaster-bearing clade, and may be phylogenetically close to *Characella* or to *Pachastrella* ^p^, both of which have small ectosomal monoaxial spicules.

### Evolution of Megascleres in the *Astrophorida* ^p^ (Fig. 3)

*Astrophorida* ^p^ species are well characterized by the simultaneous presence of asters (microscleres) and triaenes (megascleres) (Fig. 1e–g). Therefore, the classification of this order has essentially been based on variants of these two spicule types. The triaene is a synapomorphy of the *Tetractinellida* ^p^ so it appeared in the common ancestor of Spirophorida and *Astrophorida* ^p^. Since then, it has evolved in different directions giving rise to numerous descriptive terms with respect to the cladome orientation (ortho/plagio/pro/meso/anatriaenes), cladome branching (phyllo/disco/dichotriaene) or the rhabdome length (long-shafted/short-shafted/pseudocalthrops/calthrops). According to our data, the presence of triaenes or anatriaenes is not likely in the common ancestor of *Astrophorida* ^p^ (Fig. 3). This is probably due to the presence of *Alectona* and *Thrombus* at the base of the tree, both without triaenes. Long-shafted triaenes possibly appear (*p* = 0.68) in the ancestor of the Theneidae and the rest of the *Astrophorida* ^p^. Since then, they have evolved into short-shafted triaenes or calthrops. Calthrops have appeared independently many times (*Calthropella* ^p^, *Pachastrella* ^p^, *Dercitus*, some *Vulcanella*), and so have mesocalthrops and mesodichotriaenes (*Calthropella* ^p^, some *Pachastrella* ^p^). Concerning anatriaenes, our analyses (Fig. 3) suggest that they have appeared independently many times (in *Thenea* ^p^, *Characella*, some *Stelletta*, *Geodinae* ^p^). Discotriaenes have appeared independently in some lithistid *Astrophorida* ^p^ (e.g. *Discodermia*) and in the larvae of *Alectona*, although we cannot rule out the possibility that they are present in other *Astrophorida* ^p^ larvae (never observed to date). Phyllotriaenes are only known in some lithistid families, but may have appeared independently at least twice (Phymaraphiniidae and *Theonella*). To conclude, most variants of triaenes are clearly the product of convergent evolution and thus homoplasic characters that cannot be used for *Astrophorida* ^p^ classification. On the other hand, they may still represent apomorphies at lower ranks.

Before going further, we should clarify the term ‘secondary loss’. An ‘absence’ state can be optimized as a plesiomorphy (true absence), a homoplasy (independent secondary losses which appeared through convergent evolution) or a synapomorphy (unique secondary loss shared by a single clade) [97]. In this last case, ‘absence’ states may also potentially bring phylogenetic information. Furthermore, a spicule secondary loss can be i) a ‘true’ loss when nothing replaces the spicule lost (e.g. loss of sterrasters) or ii) a ‘semantic’ loss by modification of a spicule into another (e.g. sterrasters becoming aspidasters). It may not always be possible to discriminate a ‘true’ loss from a ‘semantic’ loss. For example, secondary loss of triaenes is ambiguous because some species may have retained megascleres derived from triaenes, such as styles while others may have really lost their triaenes. We therefore considered that when styles were present, it was a semantic loss, because when only oxeas remained it had a higher chance of being a true loss of triaenes.

Our study shows that triaenes have been secondarily lost (with *p*>0.65) independently at least four times in our sampling (e.g. *Melophlus*, *Asteropus*, *Vulcanella (Annulastrella)*, *Neamphius*) and morphology suggests that it may have happened in even more Astrophorida taxa, not all sampled here (*Thrombus*, *Lamellomorpha*, *Holoxea*, *Jaspis*, some *Stelletta*, some *Rhabdastrella*, some *Erylus*, some *Geodia*) [62], [78], [83], [98]. We observe similar results for anatriaenes which may have been lost eight times independently. It is also worth mentioning that anatriaenes do not seem to have been lost in the *Erylinae* ^p^ as suggested before [35]. According to our results (Fig. 3), the common ancestor of the *Geodiidae* ^p^ did not have anatriaenes, they only seem to appear in the *Geodinae* ^p^. Their absence should therefore not be considered as a synapomorphy of the *Erylinae* ^p^ [35] but as a plesiomorphy.

Our results clearly demonstrate how common secondary loss of a megasclere is, even when this megasclere has a clear function: providing support of the cortex, organization of the choanosome or even defending against predators. Secondary loss of triaene is a homoplasic character for the *Astrophorida* ^p^, but it may become synapomorphic in more restricted clades (e.g. *Vulcanella (Annulastrella)*, *Melophlus*). Also, we remind that loss of triaenes can be “partial” if it takes place during the development (e.g. *Alectona*) so increasing our knowledge in *Astrophorida* ^p^ larvae may shed some light on the classification and the evolution of triaenes.

### Evolution of Microscleres in the *Astrophorida* ^p^ (Fig. 4)

Thrombidae species have a unique type of amphiaster with recurved spines at each end, not found anywhere else in the *Astrophorida* ^p^. It has been secondarily lost in some species of *Thrombus*. It is unclear if their amphiasters are homologous to the more typical amphiasters observed in *A. millari*. Thrombidae also have trichotriaenes, not found anywhere else in the *Astrophorida* ^p^. Since trichotriaenes are fairly small (compared to true triaenes) and coexist with true triaenes in *Yucatania*; they may be derived from a large microsclere, and are certainly not triaenes *per se*. Seemingly, in *Thenea* ^p^ and *Vulcanella* (*Annulastrella*) large plesiasters have occasionally been considered as megascleres [81]. Trichotriaenes could therefore have originated from a form of plesiaster. The characteristic large diactines in *Alectona* are also thought to be derived from large asters [17]. Supporting this hypothesis are the large triactines found in some *Alectona* and the oxyasters found in *Thoosa*. However, according to the position of *A. millari* in our tree, and if we are right about the reallocation of *Thoosa* with *Alectona*, these oxyasters are not homologous to the ones that appeared later in the *Ancorinidae* ^p^ and the *Geodiidae* ^p^. As for the fusiform amphiasters found in *Alectona*, their origin remains unknown. Meanwhile, the diversified streptaster set (spirasters, metasters, plesiasters) that developed in the Theneidae and *Vulnellidae* ^p^ may have been reduced to amphiasters in the ancestor of Clade A. On one side, the *Ancorinidae* ^p^ share a close common ancestor with the lithistids/*Characella*/*Neamphius*. On the other side, the *Geodiidae* ^p^ share a close common ancestor with the newly defined Pachastrellidae. In both cases, we can hypothesize that a shortening and disappearance of the shaft and/or compression of amphiaster, spirasters or even sanidasters could have easily led to the appearance of euasters. Indeed, such ‘intermediate’ forms of asters can be observed in *Characella*, *Pachastrella* [99], *Dercitus* [73] or *Neophrissospongia* [32]. Two independent appearances of euasters in the *Astrophorida* ^p^ are not surprising in comparison with their independent appearance in *Thoosa*, some Hadromerida and in *Chondrilla* (Chondrosida). The reversed evolution is also known: amphiasters are derived from euasters in the case of *Erylus amphiastera* from Colombia (not sampled). According to our data, sterrasters have appeared once (*p*>0.65) in the ancestor of the *Geodiidae* ^p^. Evolution of spherules seem to be possible from microrhabds (as in *Caminus* [35]) or from asters (as in some *Calthropella* [73]). The sanidasters may have evolved from amphiasters and/or microrhabds but our spicule reconstructions do not support this at the moment (Fig. 4). We have nonetheless observed sanidaster-like amphiasters (in *Pachastrella abyssi*) and sanidaster-like microrhabds (in some *Pachymatisma* ^p^ *normani*). We must stress that the intermediate nodes leading to the *Ancorinidae* ^p^ and the *Geodiidae* ^p^ are poorly supported so these hypotheses need to be tested with additional molecular markers. The origin of microrhabds is seemingly contentious. The limit between microxeas, sanidasters and microrhabds is ambiguous and probably reflects their multiple appearances. They have independently appeared in (some) *Ecionemia*, *Pachastrella* ^p^, the *Erylinae* ^p^, some lithistids and *Characella* (if we consider that small microxeas present in the cortex are microrhabds). In some cases, such as in the *Erylinae* ^p^, they might be derived from asters [35]. The appearance of microxeas in the ancestor of the *Vulcanellidae* ^p^ might also be linked to asters. In the Theneidae, plesiasters reduced to two actines are common: they look like microxeas and are usually larger than the rest of the plesiasters. This is well documented in *Vulcanella* (*Annulastrella*) [37], [89] and *Thenea* ^p^ [100], [101], [102], [103], so we suggest that the microxeas found in the *Vulcanellidae* ^p^ (and maybe later in the lithistids, *Pachastrella* ^p^ and *Characella*) may have originated from large plesiasters reduced to two actines.

Sterrasters have been secondarily lost at least nine times independently (*p*>0.95) (Fig. 4): in *Penares* ^p^, *Erylus* ^p^ sp., *Erylus* ^p^ *candidata*, *Melophlus* sp., *Geostelletta* ^p^, *Calthropella* ^p^, *E. megastylifera*, *R. globostelleta+Rhabdastrella* sp. and *R. intermedia*. This clearly demonstrates how common secondary loss of a microsclere is, even when it has a clear function (sterrasters form a strong barrier protecting the sponge). Interestingly, most of the secondary losses of sterrasters have occurred in shallow-water species, living in tropical or temperate — never boreal or arctic — waters (Fig. 4). Actually, our results suggest that secondary loss of megascleres and microscleres are more common in shallow-water species. It is therefore tempting to propose that secondary loss of spicules has been favored in tropical to temperate shallow-waters. This further suggests that environmental parameters such as lower pressure, higher water temperature and/or lower silica concentration could be responsible for the loss of these sterrasters. Such parameters are already known for their effect on spicule morphology [104], [105], [106], [107], especially silica concentration that appears to have played an important role in sponge evolution [108], [109]. But since there is insufficient evidence for our hypotheses, we refrain from further speculation along these lines.

### Conclusion

This study is the first comprehensive molecular phylogenetic study of the Astrophorida. We obtained a well-resolved tree that suggested phylogenetic relationships between 89 species of Astrophorida from nine families of sponges. Most incongruences found between the current classification (*Systema Porifera*) and our molecular tree systematically made sense in the light of morphology (e.g. reallocated Ancorinidae, *G. intuta*, *D. bucklandi*, *C. pachastrelloides*), scattered biochemical data and homoplasic processes (convergent evolution and secondary loss). The taxonomic translation of this tree was a revision of the Astrophorida for which we proposed new classifications: the Linnaean classification includes all extant taxa belonging to the Astrophorida (File S2) while the phylogenetic classification includes at the moment only clades supported by molecular data and morphological data (File S1, Fig. 5). We propose in File S3 a key to all the Astrophorida families, sub-families and genera *incertae sedis*. And Table S4 summarizes the nomenclatural changes resulting from our study with respect to the name of Astrophorida species. With addition of the eight families of lithistids as well as the Thoosidae and *Neamphius huxleyi*, the Astrophorida became a larger order than previously considered, comprising ca 820 species [6]. However, the phylogenetic position of a few Astrophorida genera not sampled here is still pending (File S2). The polyphyly of some genera (*Ecionemia*, *Rhabdastrella*, *Erylus*, *Stelletta*) suggest that they should be tested on a species to species basis. Finally, other contentious groups need to be tested as potential members of the *Astrophorida* ^p^: some may have been confused with aster-bearing Hadromerida (e.g. *Jaspis* vs. *Hemiasterella*) while others may have lost all their asters and triaenes and are mixed in polyphyletic orders such as the Halichondrida or Haplosclerida.

Our study is far from being the first study to show the potential misleading nature of spicules and to question their utility in sponge taxonomy [22], [110], [111], [112], especially with the numerous studies on the phenotypical plasticity of spicules (e.g. [113]) and the recent outburst of cryptic species identification [114], [115], [116]. But this is certainly the first study to show how widespread convergent evolution and secondary loss can be in spicule evolution: they have taken place many times, in all taxa, in megascleres and microscleres, even when these seem to be adaptative and under selective pressures. Our results show for the first time the banality of spicule secondary loss (especially for microscleres) and its potential as a synapomorphy (e.g. in *Geostelletta* ^p^). With a sponge classification depending so much on spicules, secondary loss of spicules should from now on be taken more into account in future research on sponge taxonomy and phylogeny.

## Supporting Information

Figure S1 Molecular phylogeny of the Astrophorida obtained with maximum likelihood analyses (metREV+G model) of the COI amino-acid dataset. Bootstrap values >50 are given at the nodes (2,000 ML replicates).
(TIF)

Figure S2 Molecular phylogeny of the Astrophorida obtained with maximum likelihood analyses (HKY+I+G model) of the COI nucleotide dataset. Bootstrap values >50 are given at the nodes (2,000 ML replicates).
(TIF)

Figure S3 Molecular phylogeny of the Astrophorida obtained with maximum likelihood analyses (GTR+I+G model) of the 28S (C1-D2) dataset. Bootstrap values >50 are given at the nodes (2,000 ML replicates).
(TIF)

Table S1 Locality of collection, museum voucher numbers and Genbank accession numbers for the sponge specimens used in this study.
(DOC)

Table S2 Sponge identification modifications after re-examination of Astrophorida specimens from previous molecular phylogenetic and biochemistry studies.
(DOC)

Table S3 Morphological matrix of the Astrophorida species sampled in this study.
(DOC)

Table S4 Nomenclatural changes in the Linnaean and phylogenetic classification as a result of our study.
(DOC)

File S1 Definition of new clades defined in this study (following the rules of the *PhyloCode v.4c*).
(DOC)

File S2 Proposal for a new Linnaean classification of the Astrophorida.
(DOC)

File S3 Key to the Astrophorida families, sub-families and genera *incertae sedis*.
(DOC)
