# Supplementary figures and images for: Molecular Phylogeny of the Astrophorida (Porifera, Demospongiae p) Reveals an Unexpected High Level of Spicule Homoplasy
Source: PLoS One. 2011 Apr 8;6(4):e18318. doi: 10.1371/journal.pone.0018318 (PMC3072971; doi:10.1371/journal.pone.0018318)

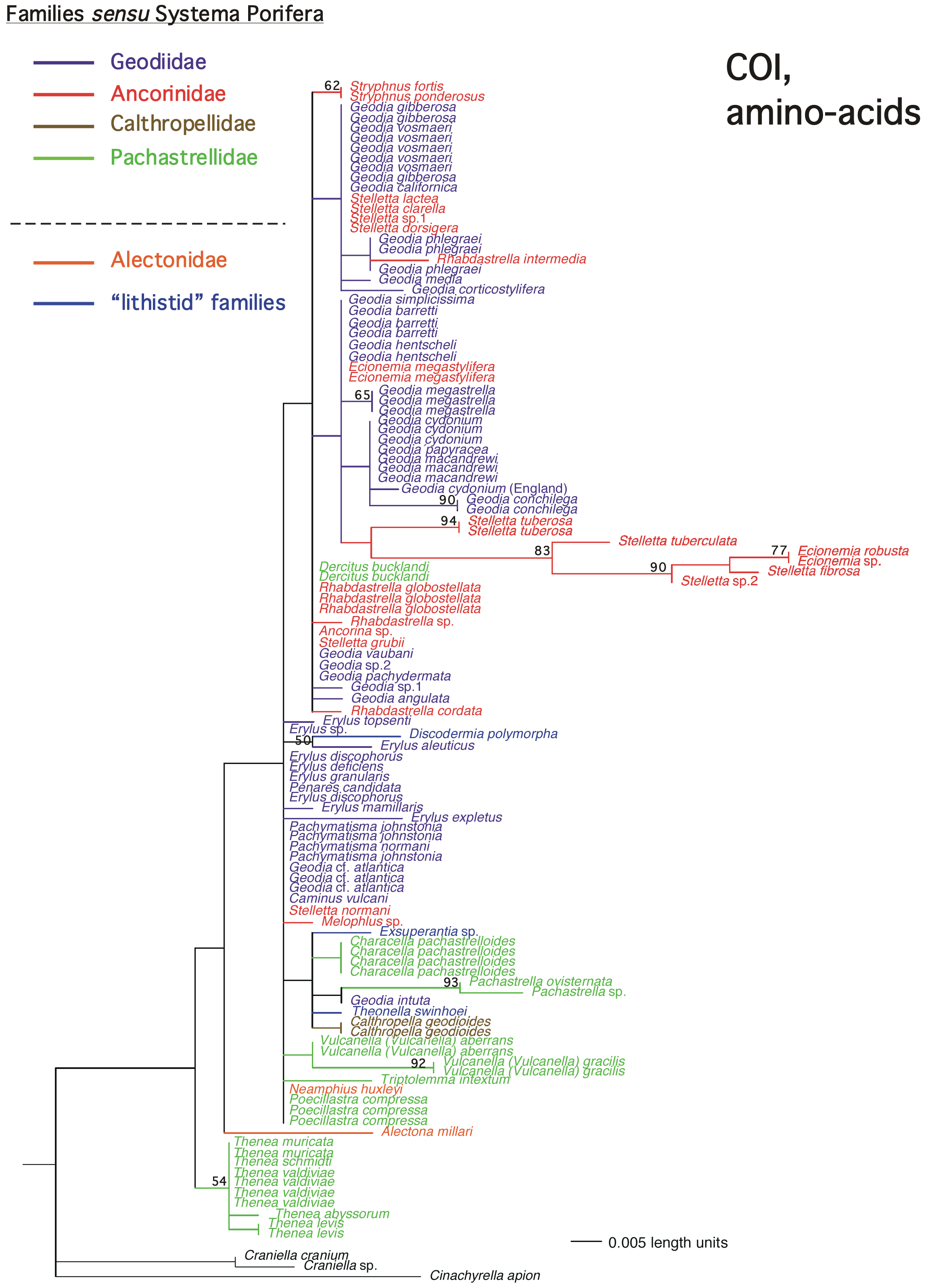

Supplement: Figure S1 — Molecular phylogeny of the Astrophorida obtained with maximum likelihood analyses (metREV+G model) of the COI amino-acid dataset. Bootstrap values >50 are given at the nodes (2,000 ML replicates). (TIF) [file pone.0018318.s001.tif]

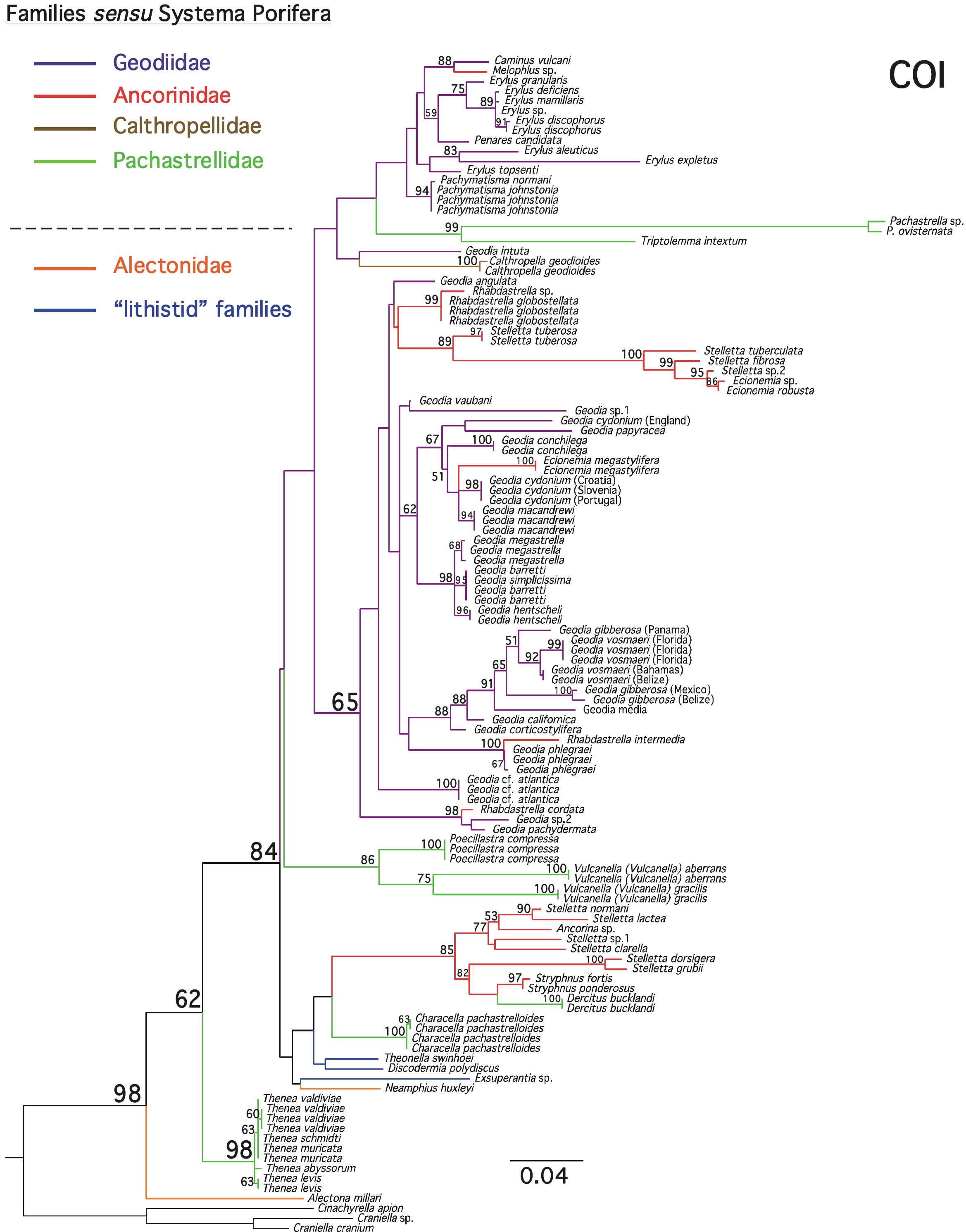

Supplement: Figure S2 — Molecular phylogeny of the Astrophorida obtained with maximum likelihood analyses (HKY+I+G model) of the COI nucleotide dataset. Bootstrap values >50 are given at the nodes (2,000 ML replicates). (TIF) [file pone.0018318.s002.tif]

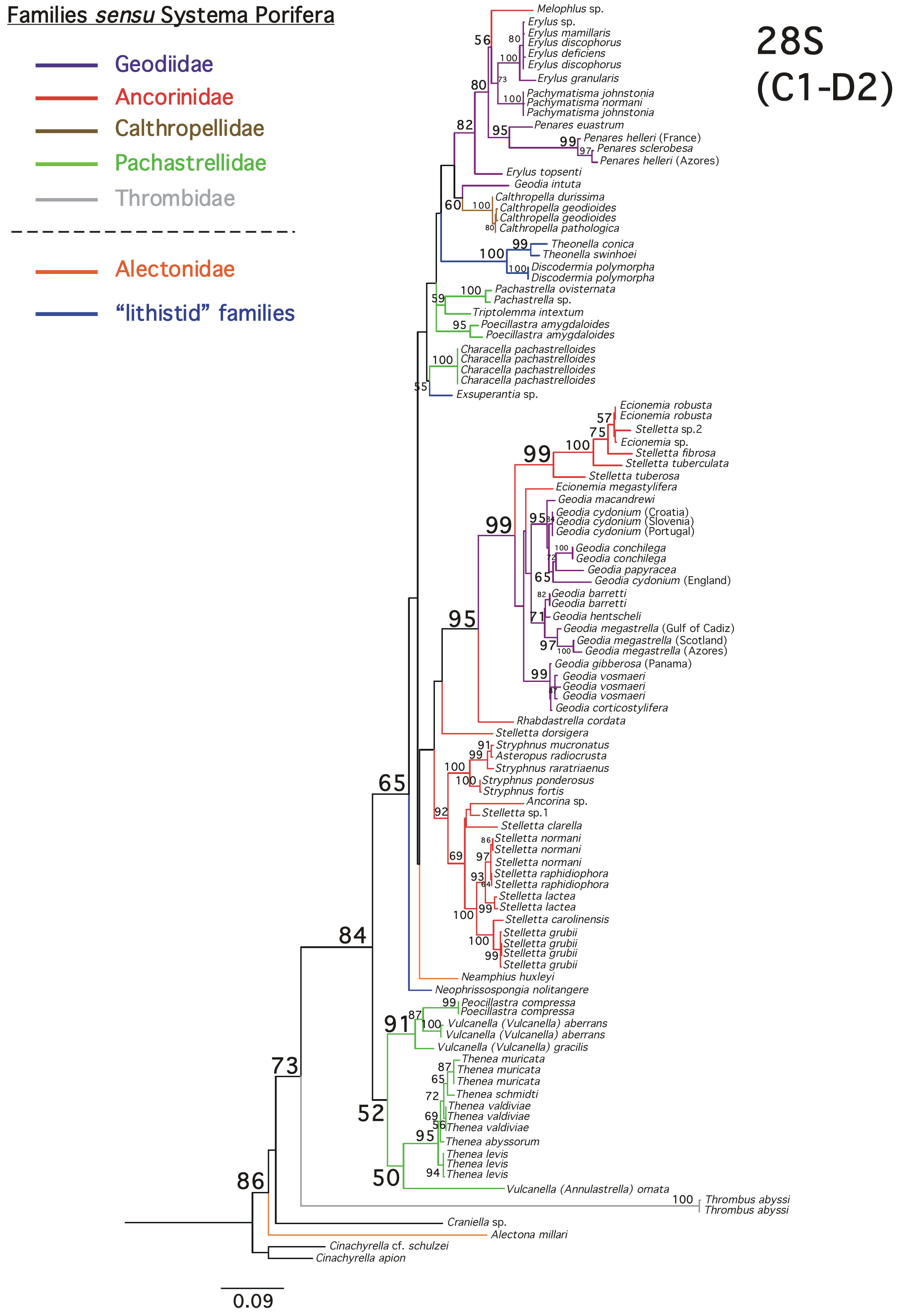

Supplement: Figure S3 — Molecular phylogeny of the Astrophorida obtained with maximum likelihood analyses (GTR+I+G model) of the 28S (C1-D2) dataset. Bootstrap values >50 are given at the nodes (2,000 ML replicates). (TIF) [file pone.0018318.s003.tif]
