# Supplementary material for: Molecular Phylogeny of the Astrophorida (Porifera, Demospongiae p) Reveals an Unexpected High Level of Spicule Homoplasy
Source: PLoS One. 2011 Apr 8;6(4):e18318. doi: 10.1371/journal.pone.0018318 (PMC3072971; doi:10.1371/journal.pone.0018318)
Supplement: Table S1 — Locality of collection, museum voucher numbers and Genbank accession numbers for the sponge specimens used in this study. (DOC) [file pone.0018318.s004.doc]

**Table S1** Locality of collection, museum voucher numbers and Genbank accession numbers for the sponge specimens used in this study. In bold, new sequences from this article.

| Species | Voucher No. | COI | 28S | Collection site |
| --- | --- | --- | --- | --- |
| **Family Geodiidae** |  |  |  |  |
| Subfamily Geodinae |  |  |  |  |
| Geodia angulata | ZMBN 77926 | EU442203 | - | Carmel, CA (U.S.A.) |
| *Geodia* cf. *atlantica* | ZMAPOR 19647 | **HM592679** | - | Rockall Bank (off Ireland) |
| *Geodia* cf. *atlantica* | ZMBN 85200 | **HM592695** | - | Røst reef (Northern Norway) |
| *Geodia* cf. *atlantica* | ZMBN 77927 | EU442195 | - | Korsfjord (Western Norway) |
| *Geodia barretti* | ZMBN 77922 | EU442194 | EU552080^f^ | Korsfjord (Western Norway) |
| *Geodia barretti* | ZMBN 85201 | **HM592684** | - | Skagerrak (Southern Norway) |
| *Geodia barretti* | ZMBN 85202 | **HM592720** | **HM592809** | Hebrides Islands (Scotland) |
| *Geodia californica* | UMPCW913 | EU442200 | - | Sea of Cortez (Mexico) |
| *Geodia conchilega* | ZMAPOR 21650 | **HM592739** | **HM592808** | Berlengas Islands (Portugal) |
| *Geodia conchilega* | ZMAPOR 21651 | **HM592742** | **HM592807** | Faial Island (Azores) |
| *Geodia corticostylifera* | ZMBN 85203 | **HM592681** | **HM592815** | Buzios (Brasil) |
| *Geodia cydonium* | ZMBN 85204 | **HM592715** | **HM592814** | Brixham, Devon (England) |
| *Geodia cydonium* | ZMAPOR 21652 | **HM592738** | **HM592806** | Berlengas (Portugal) |
| *Geodia cydonium* | ZMAPOR 21439 | **HM592693** | **HM592805** | Piran (Slovenia) |
| *Geodia cydonium* | ZMBN 77923 | EU442199 | EU552081^f^ | Rovinj (Croatia) |
| *Geodia gibberosa* ^a^ | CNPGG-0078 | **HM592723** | - | North Yucatan (Mexico) |
| *Geodia gibberosa* ^a^ | ZMBN 77928 | EU442209 | FJ717708 | Bocas del Toro (Panama, Atlantic) |
| *Geodia gibberosa* ^a^ | - | EF519614 | - | South Reef (Belize) |
| Geodia hentscheli | ZMBN 85205 | **HM592671** | - | Northern Iceland Plateau |
| Geodia hentscheli | ZMBN 77925 | EU442197 | EU552083^f^ | ‘The Schultz Massive’ seamount (Greenland Sea) |
| *Geodia intuta* | ZMAPOR 21653 | **HM592740** | **HM592804** | Berlengas Islands (Portugal) |
| *Geodia macandrewi* | ZMBN 77924 | EU442198 | EU552082^f^ | Korsfjord (Western Norway) |
| *Geodia macandrewi* | ZMBN 85206 | **HM592689** | - | Hjeltefjord (Western Norway) |
| *Geodia macandrewi* | ZMBN 85207 | **HM592696** | - | Røst reef (Northern Norway) |
| *Geodia media* | UMPCW927 | AY561962 | - | Isla Contadora (Panama, Pacific) |
| Geodia megastrella | ZMBN 85208 | - | **HM592810** | Hebrides Islands (Scotland) |
| Geodia megastrella | ZMBN 85209 | **HM592721** | - | Hebrides Islands (Scotland) |
| Geodia megastrella | ZMAPOR 21654 | **HM592731** | **HM592811** | Seamounts south of Azores |
| Geodia megastrella | ZMAPOR 21231 | **HM592741** | **HM592812** | Gulf of Cadiz (off Morocco) |
| Geodia pachydermata | ZMAPOR 21655 | **HM592732** | - | Seamounts south of Azores |
| Geodia papyracea | UMPCW921 | AY561961 | FJ717707 | Bocas del Toro (Panama, Caribbean) |
| Geodia phlegraei | ZMBN 85210 | **HM592690** | - | ‘The Schultz Massive’ seamount (Greenland Sea) |
| Geodia phlegraei | ZMBN 85211 | **HM592701** | - | Trænadjupet (Northern Norway) |
| Geodia phlegraei | ZMBN 77929 | EU442196 | - | Korsfjord (Western Norway) ^b^ |
| Geodia simplicissima | ZMBN 85212 | **HM592691** | - | Trellholmstetta (Western Norway) |
| Geodia vaubani | IRD-NC-R1822 | EU442202 | - | Stylaster bank (New Caledonia) |
| *Geodia vosmaeri* | - | EF519674 | - | Tennessee Reef, Florida Keys, FL (U.S.A.) |
| *Geodia vosmaeri* | - | AY320032 | EU552086 ^d^ | Tennessee Reef, Florida keys, FL (U.S.A.) |
| Geodia vosmaeri | ZMBN 85213 | **HM592722** | **HM592817** | Key Largo, Florida keys, FL (U.S.A.) |
| Geodia vosmaeri | ZMBN 85214 | **HM592711** | **HM592816** | Sweetings Cay (Bahamas) |
| *Geodia vosmaeri* | - | EF519673 | - | Fire Carrie Bow Reef (Belize) |
| Geodia sp. 1 | IRD-NC-R1820 | **HM592680** | - | Stylaster bank (New Caledonia) |
| *Geodia* sp. 2 | MNHN DCL4075 | **HM592707** | - | off Cape S. Maria di Leuca (Southern Italy) |
| Subfamily Erylinae |  |  |  |  |
| *Caminus vulcani* ^a^ | ZMAPOR 20422 | EU442205 | - | Tenerife (Canary Islands) |
| *Pachymatisma normani* | ZMBN 77858 ^c^ | EF564322 | EU552087^f^ | Korsfjord (Western Norway) ^b^ |
| *Pachymatisma johnstonia* ^a^ | ZMAPOR 20348a | EF564330 | - | Mingulay reef (Scotland) |
| *Pachymatisma johnstonia* ^a^ | MNHN DCL4015 | EF564335 | AF062601 | Roscoff (France) |
| *Pachymatisma johnstonia* ^a^ | ZMAPOR 21442 | EF564338 | **HM592832** | Berlengas Islands (Portugal) |
| *Erylus aleuticus* | ZMBN 77933 ^c^ | EU442201 | - | Amlia Island, AL (U.S.A.) ^b^ |
| *Erylus deficiens* | ZMAPOR 20419 | EU442204 | EU552088^f^ | Reserva do Garajau (Madeira Islands) |
| *Erylus discophorus* | ZMAPOR 20420 | EU442206 | EU552089^f^ | Berlengas Islands (Portugal) |
| *Erylus discophorus* | ZMAPOR 21716 | **HM592692** | **HM592822** | Piran (Slovenia) |
| *Erylus expletus* | ZMAPOR 18142 | EU442208 | - | South East of Rockall Bank (Ireland) |
| *Erylus granularis* | ZMAPOR 21656 | **HM592729** | **HM592827** | Seamounts south of Azores |
| *Erylus mamillaris* ^a^ | ZMAPOR 20421 | EU442207 | EU552090^f^ | Faial Island (Azores) |
| *Erylus topsenti* | ZMAPOR 21657 | **HM592733** | **HM592831** | Seamounts south of Azores |
| *Erylus* sp. | ZMAPOR 21693 | **HM592687** | **HM592823** | Gettysburg Peak, Gorringe Bank |
| *Penares candidata* | ZMAPOR 21440 | **HM592719** | - | Berlengas Islands (Portugal) |
| *Penares euastrum* | MNHN DCL4069 | - | AF062600 | Banc de l’Esquine (France, Mediterranean Sea) |
| *Penares helleri* ^a^ | MNHN DCL4068 | - | AF062598 | La Ciotat (France, Mediterranean Sea) |
| *Penares helleri* ^a^ | ZMAPOR 21658 | - | **HM592828** | Flores Island (Azores) |
| *Penares sclerobesa* | ZMAPOR 21659 | - | **HM592829** | Seamounts south of Azores |
| **Family Ancorinidae** |  |  |  |  |
| *Ancorina* sp. | ZMAPOR 21660 | **HM592744** | **HM592785** | Gorringe Bank |
| *Asteropus radiocrusta* | S1013 | - | **HM592784** | Investigator group Island (South Australia) |
| *Ecionemia megastylifera* | UCMPWC980 | AY561938 | - | Bocas del Toro (Panama, Caribbean) |
| *Ecionemia megastylifera* | ZMBN 81782 | FJ711642 | FJ711648 | Bocas del Toro (Panama, Caribbean) |
| Ecionemia robusta | S1017 | - | **HM592801** | Investigator group Island (South Australia) |
| Ecionemia robusta | S1018 | **HM592724** | **HM592802** | Investigator group Island (South Australia) |
| *Ecionemia* sp. | S1020 | **HM592725** | **HM592803** | Investigator group Island (South Australia) |
| Melophlus sp. | UCMPWC1052 | **HM592688** | **HM592821** | Baluan Island (Papua New Guinea) |
| *Rhabdastrella cordata* | S1026 | **HM592727** | **HM592813** | Investigator group Island (South Australia) |
| Rhabdastrella globostellata | USP 9712SD114 | **HM592673** | - | Vanua Levu Island (Fiji Islands) |
| *Rhabdastrella globostellata* | UCMPWC1072 | **HM592683** | - | Manus Island (Papua New Guinea) |
| Rhabdastrella globostellata | ZMAPOR 17240 | **HM592746** | - | Shelly Beach, off Mombassa (Kenya) |
| *Rhabdastrella intermedia* | S1025 | **HM592726** | - | Investigator group Island (South Australia) |
| Rhabdastrella sp. | PDZ_1_ 98-1-10 | **HM592676** | - | Mindanao (Philippines) |
| *Stelletta carolinensis* | ZMAPOR 17977 | - | **HM592798** | Off Georgia (U.S.A.) |
| *Stelletta clarella* | ZMAPOR 21673 | **HM592736** | **HM592797** | Monterey Bay, CA (U.S.A.) |
| *Stelletta dorsigera* | MNHN DCL4070 | **HM592750** | AY348892 | Les Roches Torreilles (France, Mediterranean Sea) |
| *Stelletta fibrosa* | ZMBN 81784 | FJ711643 | FJ711649 | Bocas del Toro (Panama, Caribbean) |
| Stelletta grubii ^a^ | Mc 2668 | - | **HM592786** | Rathlin Island (Northern Ireland) |
| Stelletta grubii ^a^ | ZMBN 85219 | - | **HM592787** | Brixham, Devon (England) |
| Stelletta grubii ^a^ | ZMAPOR 21661 | **HM592743** | - | Berlengas Islands (Portugal) |
| Stelletta grubii ^a^ | ZMAPOR 21662 | - | **HM592789** | Berlengas Islands (Portugal) |
| Stelletta grubii ^a^ | ZMAPOR 21663 | - | **HM592788** | Berlengas Islands (Portugal) |
| Stelletta lactea | Mc4945 | **HM592752** | **HM592795** | Strangford Lough (Northern Ireland) |
| Stelletta lactea | ZMAPOR 21664 | - | **HM592794** | Seamounts south of Azores |
| *Stelletta normani* | ZMBN 85220 | - | **HM592792** | Sotbakken (Northern Norway) |
| Stelletta normani | ZMBN 77930 | EU442193 | - | Langenuen (Western Norway) |
| *Stelletta normani* | ZMBN 77931 | - | EU55209 | Korsfjord (Western Norway) ^b^ |
| *Stelletta normani* | ZMBN 85221 | - | **HM592793** | Hebrides Islands (Scotland) |
| *Stelletta raphidiophora* | ZMBN 85222 | - | **HM592790** ^d^ | Northern Iceland Plateau |
| *Stelletta raphidiophora* | ZMBN 85223 | - | **HM592791** | ‘The Schultz Massive’ seamount (Greenland Sea) |
| *Stelletta tuberosa* | MNHN DCL4066 | **HM592678** | - | Bay of Biscay (Northern Spain) |
| *Stelletta tuberosa* | ZMAPOR 21665 | **HM592735** | **HM592799** | Seamounts south of Azores |
| *Stelletta tuberculata* | S1027 | **HM592728** | **HM592800** | Investigator group Island (South Australia) |
| *Stelletta* sp. 1 | Mc4777 | **HM592751** | **HM592796** | South Grand Jason (Falkland Islands) |
| *Stelletta* sp. 2 | ZMBN 81643 | FJ711644 | FJ711650 | Bocas del Toro (Panama, Caribbean) |
| *Stryphnus fortis* | ZMBN 85224 | - | **HM592782** ^d^ | Korsfjord (Western Norway) |
| *Stryphnus fortis* | ZMBN 82977 | **HM592697** | - | Korsfjord (Western Norway) |
| *Stryphnus mucronatus* | MNHN DCL4071 | - | AF062597 | La Ciotat (France, Mediterranean Sea) |
| *Stryphnus ponderosus* | Mc3395 | **HM592685** | **HM592783** ^d^ | Rathlin Island (Northern Ireland) |
| *Stryphnus raratriaenus* | ZMBN 81642 ^c^ | - | FJ711647 | Bocas del Toro (Panama, Caribbean) |
| **Family Calthropellidae** |  |  |  |  |
| *Calthropella durissima* | ZMAPOR 21666 | - | **HM592824** | Seamounts south of Azores |
| *Calthropella geodioides* | MNHN DCL4076 | **HM592705** | **HM592826** | off Cape S. Maria di Leuca (Southern Italy) |
| Calthropella geodioides | ZMAPOR 21667 | **HM592734** | **HM592825** | Seamounts south of Azores |
| Calthropella pathologica | MNHN DCL4067a | - | AF062596 | La Ciotat (France, Mediterranean Sea) |
| **Family Pachastrellidae** |  |  |  |  |
| Characella pachastrelloides | ZMBN 80248 | **HM592672** | **HM592778** | Hjeltefjord (Western Norway) |
| *Characella pachastrelloides* | ZMAPOR 20375 | **HM592749** | **HM592781** | Mingulay Reef, Scotland (United Kingdom) |
| *Characella pachastrelloides* | ZMBN 85225 | **HM592709** | **HM592780** | Setúbal Canyon (Portugal) |
| Characella pachastrelloides | ZMAPOR 18041 | **HM592713** | **HM592779** | Gulf of Cadiz (Spain) |
| Dercitus bucklandi ^a^ | Mc2649 | **HM592674** | - | Rathlin Island (Northern Ireland) |
| *Dercitus bucklandi* ^a^ | ZMBN 85226 | **HM592716** | - | Brixham, Devon (England) |
| *Pachastrella* sp. | ZMBN 85227 | **HM592698** | **HM592775** | Korsfjord (Western Norway) |
| Pachastrella ovisternata | ZMAPOR 21219 | **HM592748** | - | Bay of Biscay (off France) |
| Pachastrella ovisternata | ZMAPOR 21224 | - | **HM592774** | Gulf of Cadiz (Off Morocco) |
| Poecillastra amygdaloides | ZMAPOR 21223 | - | **HM592773** | Gulf of Cadiz (Off Morocco) |
| Poecillastra amygdaloides | MNHN DCL4077 | - | **HM592772** | off Cape S. Maria di Leuca (Southern Italy) |
| Poecillastra compressa ^a^ | ZMBN 77932 | EU442192 | - | Langenuen (Western Norway) |
| *Poecillastra compressa* ^a^ | ZMBN 85251 | - | **HM592757** | Hjeltefjord (Western Norway) |
| *Poecillastra compressa* ^a^ | MNHN DCL4072 | **HM592714** | AF062599 | Banc de l’Esquine (France, Mediterranean Sea) |
| *Poecillastra compressa* ^a^ | ZMBN 86300 | **HM592675** | - | Rockall Bank (off Ireland) |
| *Thenea abyssorum* | ZMBN 85228 | **HM592712** | **HM592770** | Greenland Sea |
| *Thenea levis* | ZMBN 85229 | - | **HM592764** | Sotbakken (Northern Norway) |
| *Thenea levis* | ZMBN 85230 | **HM592717** | **HM592765** | Off Korsfjord (Western Norway) |
| *Thenea levis* | ZMAPOR 21501 | **HM592747** | **HM592766** | South West of Rockall Bank (off Ireland) |
| *Thenea muricata* ^a^ | ZMBN 85231 | - | **HM592768** | off Korsfjord (Western Norway) |
| *Thenea muricata* ^a^ | ZMBN 85232 | **HM592677** | - | Brattholmen (Western Norway) |
| *Thenea muricata* ^a^ | - | - | AY552019 ^e^ | Perseverance Mound (off Ireland) |
| *Thenea muricata* ^a^ | MNHN DCL4083 | **HM592706** | **HM592767** | off Cape S. Maria di Leuca (Southern Italy) |
| *Thenea schmidti* | ZMAPOR 18036 | **HM592737** | **HM592769** | Gulf of Cadiz |
| *Thenea valdiviae* | ZMBN 85233 | **HM592708** | - | Barents Sea |
| *Thenea valdiviae* | ZMBN 85234 | **HM592694** | **HM592761** | ‘The Schultz Massive’ seamount (Greenland Sea) |
| *Thenea valdiviae* | ZMBN 85235 | **HM592703** | **HM592762** | Trænadjupet (Northern Norway) |
| *Thenea valdiviae* | ZMBN 85236 | **HM592718** | **HM592763** | off Korsfjord (Western Norway) |
| *Triptolemma intextum* | MNHN DCL4080 | **HM592710** | **HM592777** | off Cape S. Maria di Leuca (Southern Italy) |
| *Vulcanella (Annulastrella) ornata* | ZMAPOR 18003 | - | **HM592771** | Off Tanger (Morocco) |
| *Vulcanella (Vulcanella) aberrans* | ZMBN 80959 | **HM592699** | **HM592758** | Sotbakken (Northern Norway) |
| *Vulcanella (Vulcanella) aberrans* | ZMAPOR 21193 | **HM592700** | - | Gulf of Cadiz (off Morocco) |
| *Vulcanella (Vulcanella) aberrans* | ZMAPOR 18012 | - | **HM592759** | Gulf of Cadiz (off Morocco) |
| *Vulcanella (Vulcanella) gracilis* | ZMAPOR 18025 | **HM592702** | - | Off Tanger (Morocco) |
| *Vulcanella (Vulcanella) gracilis* | MNHN DCL4082 | **HM592704** | **HM592760** | off Cape S. Maria di Leuca (Southern Italy) |
| **Family Thrombidae** |  |  |  |  |
| *Thrombus abyssi* | ZMAPOR 20356 | - | **HM592756** | Mingulay Reef, Scotland (United Kingdom) |
| *Thrombus abyssi* | ZMAPOR 18227 | - | **HM592755** | Rockall Bank (off Ireland) |
| **Family Theonellidae** |  |  |  |  |
| *Discodermia polymorpha* | ZMBN 85237 | **HM592686** | **HM592819** | La Ciotat (France, Mediterranean Sea) |
| *Discodermia polymorpha* | - | - | AF062603 | La Ciotat (France, Mediterranean Sea) |
| *Theonella conica* | UCMPWC1025 | - | **HM592818** | near Selapiu Island (Papua New Guinea) |
| *Theonella swinhoei* ^a^ | ZMAPOR 16637 | **HM592745** | **HM592820** | Hurghada (Egypt) |
| **Family Corallistidae** |  |  |  |  |
| *Neophrissospongia nolitangere* ^a^ | MNHN DJV21 | - | AF062602 | La Ciotat (France, Mediterranean Sea) |
| **Family Phymaraphiniidae** |  |  |  |  |
| *Exsuperantia* sp. | ZMAPOR 21668 | **HM592730** | **HM592830** | Seamounts south of Azores |
| **Family Alectonidae** |  |  |  |  |
| *Alectona millari* ^a^ | ZMBN 85238 | **HM592670** | - | Sotbakken (Northern Norway) |
| *Alectona millari* ^a^ | - | - | AY552020 ^e^ | Perseverance Mound (off Ireland) |
| *Neamphius huxleyi* ^a^ | UCMPWC1086 | **HM592682** | **HM592776** | Manus Island (Papua New Guinea) |
| **Outgroups** |  |  |  |  |
| **Spirophorida - Tetillidae** |  |  |  |  |
| *Craniella cranium* | ZMBN 85239 | **HM592669** | - | Korsfjord (Western Norway) |
| *Craniella* sp. | ZMBN 85240 | **HM592668** | **HM592754** | Korsfjord (Western Norway) |
| *Cinachyrella apion* | ZMBN 81789 | **HM592667** | **HM592753** | Key Largo, FL (U.S.A.) |
| Cinachyrella cf. schulzei | MNHN DCL4073 | - | AF062604 | New Caledonia |

^a^ Type species of genera.

^b^ Type locality of species.

^c^ Type material.

^d^ Shorter sequences (primer Ep1a’ used).

^e^ C1-D1 sequences [1].

^f^ 28S sequences updated since Cárdenas et al. (2010) [35] by the addition of the sequence of the C1 domain.

Abbreviations: CNPGG: Colección Nacional del Phylum Porifera Gerardo Green, Instituto de Ciencias del Mar y Limnología, Universidad Nacional Autónoma de México, México; Mc, National Museums, Northern Ireland, Holywood; MNHN, Muséum National d’Histoire Naturelle, Paris; IRD-NC, Institut de Recherche pour le Développement, Nouméa, Nouvelle-Calédonie; PDZ, University of Utah, Salt Lake City; S, South Australian Museum, Adelaide; UCMPW, University of California Museum of Paleontology, Berkeley, CA; USP, Regional Herbarium, School of Pure and Applied Sciences, University of the South Pacific, Fiji; ZMA, Zoölogisch Museum van de Universiteit van Amsterdam; ZMBN, Bergen Museum.
