# Supplementary material for: Molecular Phylogeny of the Astrophorida (Porifera, Demospongiae p) Reveals an Unexpected High Level of Spicule Homoplasy
Source: PLoS One. 2011 Apr 8;6(4):e18318. doi: 10.1371/journal.pone.0018318 (PMC3072971; doi:10.1371/journal.pone.0018318)
Supplement: Table S2 — Sponge identification modifications after re-examination of Astrophorida specimens from previous molecular phylogenetic and biochemistry studies. (DOC) [file pone.0018318.s005.doc]

**Table S2** New Astrophorida identification after re-examination of specimens from previous molecular phylogenetic and biochemistry studies.

| **Original identification** | **Authorship of identification** | **Voucher** | **New identification** | **Authorship  of new identification** |
| --- | --- | --- | --- | --- |
| *Corallistes masoni* | [5] | MNHN DJV21 | *Neophrissospongia nolitangere* | [32] |
| *Discodermia polydiscus* | [5] | - | *Discodermia polymorpha* | [32] |
| *Stelletta grubei* | [24], [1] | MNHN DCL4070 | *Stelletta dorsigera* | P. Cárdenas |
| *Holoxea* sp. | [4] | UCMPWC 1025 | *Theonella conica* | P. Cárdenas |
| cf. *Lamellomorpha* sp. | [4] | UCMPWC 1086 | *Neamphius huxleyi* | P. Cárdenas |
| *Ecionemia* sp. | [4] | UCMPWC 980 | *Ecionemia megastylifera* | [34] |
| *Rhabdastrella* sp. | [4] | UCMPWC 1072 | *Rhabdastrella globostellata* | P. Cárdenas |
| *Geodia globostellifera* | [30] | USP 9712SD114 | *Rhabdastrella globostellata* | [31] |
| *Rhabdastrella globostellata* | [31] | PDZ_1_ 98-1-10 | *Rhabdastrella* sp. | P. Cárdenas |
| *Geodia* sp. | [28] | ZMBN 85213 | *Geodia vosmaeri* | P. Cárdenas |
| *Asteropus sp.* | [29] | S1013 | *Asteropus radiocrusta* | P. Cárdenas |
| *Ecionemia sp.* 1 | [29] | S1017, S1018 | *Ecionemia robusta* | P. Cárdenas |
| *Rhabdastrella sp.* 1 | [29] | S1025 | *Rhabdastrella intermedia* | P. Cárdenas |
| *Rhabdastrella sp.* 2 | [29] | S1026 | *Rhabdastrella cordata* | P. Cárdenas |

Abbreviations: MNHN, Muséum National d’Histoire Naturelle, Paris; PDZ, University of Utah, Salt Lake City; S, South Australian Museum, Adelaide ; UCMPW, University of California Museum of Paleontology, Berkeley, CA; USP, Regional Herbarium, School of Pure and Applied Sciences, University of the South Pacific, Fiji.
