# Supplementary material for: Molecular Phylogeny of the Astrophorida (Porifera, Demospongiae p) Reveals an Unexpected High Level of Spicule Homoplasy
Source: PLoS One. 2011 Apr 8;6(4):e18318. doi: 10.1371/journal.pone.0018318 (PMC3072971; doi:10.1371/journal.pone.0018318)
Supplement: Table S4 — Nomenclatural changes in the Linnaean and phylogenetic classification as a result of our study. (DOC) [file pone.0018318.s007.doc]

**Table S4** Nomenclatural changes in the Linnaean and phylogenetic classification as a result of our study.

| Species (Linnean Classification before our study) | Species (Linnean Classification after our study) | *PhyloCode* species |
| --- | --- | --- |
| **Family Geodiidae** | **Family Geodiidae** |  |
| Subfamily Geodinae | Subfamily Geodinae |  |
| Geodia angulata (Lendenfeld, 1910) | Geodia angulata | Geodinae^p^ angulata |
| *Geodia* cf. *atlantica* (Stephens, 1915) | *Geodia* cf. *atlantica* | *Geodinae*^p^ cf. *atlantica* |
| *Geodia barretti* Bowerbank, 1858 | *Geodia barretti* | *Depressiogeodia*^p^ *barretti* |
| *Geodia californica* (Lendenfeld, 1910) | *Geodia californica* | *Geodia*^p^ *californica* |
| *Geodia conchilega* Schmidt, 1862 | *Geodia conchilega* | *Cydonium*^p^ *conchilega* |
| *Geodia corticostylifera* Hajdu et al., 1992 | *Geodia corticostylifera* | *Geodia*^p^ *corticostylifera* |
| *Geodia cydonium* (Jameson, 1811) | *Geodia cydonium* | *Cydonium*^p^ *cydonium* |
|  | *Geodia* aff. *cydonium* | *Cydonium*^p^ aff. *cydonium* |
| *Geodia gibberosa* Lamarck, 1815 | *Geodia gibberosa* | *Geodia*^p^ *gibberosa* |
|  | *Geodia tumulosa* Bowerbank, 1872 | *Geodia*^p^ *tumulosa* |
| Geodia hentscheli Cárdenas et al., 2010 | Geodia hentscheli | Depressiogeodia^p^ hentscheli |
| *Geodia intuta* (Topsent, 1892) | *Caminella intuta* | *Erylinae*^p^ *intuta* |
| *Geodia macandrewi* Bowerbank, 1858 | *Geodia macandrewi* | *Cydonium*^p^ *macandrewi* |
| *Geodia media* Bowerbank, 1873 | *Geodia media* | *Geodia*^p^ *media* |
| Geodia megastrella Carter, 1876 | Geodia megastrella | Depressiogeodia^p^ megastrella |
| Geodia pachydermata (Sollas, 1886) | Geodia pachydermata | Synops^p^ pachydermata |
| Geodia papyracea Hechtel, 1965 | Geodia papyracea | Cydonium^p^ papyracea |
| Geodia phlegraei (Sollas, 1880) | Geodia phlegraei | Geodinae^p^ phlegraei |
| Geodia simplicissima Burton, 1931 | Geodia simplicissima | Depressiogeodia^p^ simplicissima |
| Geodia vaubani Lévi & Lévi, 1983 | Geodia vaubani | Geodinae^p^ vaubani |
| *Geodia vosmaeri* (Sollas, 1886) | *Geodia vosmaeri* | *Geodia*^p^ *vosmaeri* |
| Geodia sp. 1 | Geodia sp. 1 | Geodinae^p^ sp. 1 |
| *Geodia* sp. 2 | *Geodia* sp. 2 | *Synops*^p^ sp. |
| Subfamily Erylinae | Subfamily Erylinae |  |
| *Caminus vulcani* Schmidt, 1862 | *Caminus vulcani* | *Erylinae*^p^ *vulcani* |
| *Pachymatisma normani* Sollas, 1888 | *Pachymatisma normani* | *Pachymatisma*^p^ *normani* |
| *Pachymatisma johnstonia* Bowerbank *in* Johnston, 1842 | *Pachymatisma johnstonia* | *Pachymatisma*^p^ *johnstonia* |
| *Erylus aleuticus* Lehnert et al., 2006 | *Erylus aleuticus* | *Erylinae*^p^ *aleuticus* |
| *Erylus deficiens* (Topsent, 1927) | *Erylus deficiens* | *Erylus*^p^ *deficiens* |
| *Erylus discophorus* (Schmidt, 1862) | *Erylus discophorus* | *Erylus*^p^ *discophorus* |
| *Erylus expletus* Topsent, 1927 | *Erylus expletus* | *Erylinae*^p^ *expletus* |
| *Erylus granularis* Topsent, 1904 | *Erylus granularis* | *Erylus*^p^ *granularis* |
| *Erylus mamillaris* (Schmidt, 1862) | *Erylus mamillaris* | *Erylus*^p^ *mamillaris* |
| *Erylus topsenti* von Lendenfeld, 1903 | *Erylus topsenti* | *Erylinae*^p^ *topsenti* |
| *Erylus* sp. | *Erylus* sp. | *Erylus*^p^ sp. |
| *Penares candidata* (Schmidt, 1868) | *Penares candidata* | *Erylus*^p^ *candidata* |
| *Penares euastrum* (Schmidt, 1868) | *Penares euastrum* | *Penares*^p^ *euastrum* |
| *Penares helleri* (Schmidt, 1864) | *Penares helleri* | *Penares*^p^ *helleri* |
| *Penares sclerobesa* Topsent, 1904 | *Penares sclerobesa* | *Penares*^p^ *sclerobesa* |
| **Family Calthropellidae** | Subfamily Calthropellinae |  |
| *Calthropella durissima* Topsent, 1892 | *Calthropella durissima* | *Calthropella*^p^ *durissima* |
| *Calthropella geodioides* (Carter, 1876) | *Calthropella geodioides* | *Calthropella*^p^ *geodioides* |
| Calthropella pathologica (Schmidt, 1868) | Calthropella pathologica | Calthropella^p^ pathologica |
| **Family Ancorinidae** |  |  |
| *Ancorina* sp. | *Ancorina* sp. | *Ancorinidae*^p^ sp. 1 |
| *Asteropus radiocrusta* Kennedy, 2000 | *Stryphnus radiocrusta* | *Stryphnus*^p^ *radiocrusta* |
| *Ecionemia megastylifera* Wintermann-Kilian & Kilian, 1984 | *Geodia megastylifera* | *Cydonium*^p^ *megastylifera* |
| Ecionemia robusta (Carter, 1883) | Geodia robusta* | Geostelletta^p^ robusta |
| *Ecionemia* sp. | *Geodia* sp. | *Geostelletta^p^* sp. 1 |
| Melophlus sp. | Melophlus sp. | Erylinae^p^ sp. |
| *Rhabdastrella cordata* Wiedenmayer, 1989 | *Geodia cordata* | *Synops*^p^ *cordata* |
| Rhabdastrella globostellata (Carter, 1883) | Geodia globostellata | Geodinae^p^ globostellata |
| *Rhabdastrella intermedia* Wiedenmayer, 1989 | *Geodia intermedia* | *Geodinae*^p^ *intermedia* |
| Rhabdastrella sp. | Geodia sp. | Geodinae^p^ sp. 2 |
| *Stelletta carolinensis* Wells et al., 1960 | *Stelletta carolinensis* | *Stelletta*^p^ *carolinensis* |
| *Stelletta clarella* de Laubenfels, 1930 | *Stelletta clarella* | *Ancorinidae*^p^ *clarella* |
| *Stelletta dorsigera* Schmidt, 1864 | *Stelletta dorsigera* | *Stelletta*^p^ *dorsigera* |
| *Stelletta fibrosa* (Schmidt, 1870) | *Geodia fibrosa* | *Geostelletta^p^ fibrosa* |
| Stelletta grubii Schmidt, 1862 | Stelletta grubii | Stelletta^p^ grubii |
| Stelletta lactea Carter, 1871 | Stelletta lactea | Dragmastra^p^ lactea |
| *Stelletta normani* Sollas, 1880 | *Stelletta normani* | *Dragmastra*^p^ *normani* |
| *Stelletta raphidiophora* Hentschel, 1929 | *Stelletta raphidiophora* | *Dragmastra*^p^ *raphidiophora* |
| *Stelletta tuberosa* (Topsent, 1892) | *Geodia tuberosa** | *Geostelletta*^p^ *tuberosa* |
| *Stelletta tuberculata* (Carter, 1886) | *Geodia tuberculata* | *Geostelletta*^p^ *tuberculata* |
| *Stelletta* sp. 1 | *Stelletta* sp. | *Ancorinidae*^p^ sp. 2 |
| *Stelletta* sp. 2 | *Geodia* sp. | *Geostelletta*^p^ sp. 2 |
| *Stryphnus fortis* (Vosmaer, 1885) | *Stryphnus fortis* | *Stryphnus*^p^ *fortis* |
| *Stryphnus mucronatus* (Schmidt, 1868) | *Stryphnus mucronatus* | *Stryphnus*^p^ *mucronatus* |
| *Stryphnus ponderosus* (Bowerbank, 1866) | *Stryphnus ponderosus* | *Stryphnus*^p^ *ponderosus* |
| *Stryphnus raratriaenus* Cárdenas et al., 2009 | *Stryphnus raratriaenus* | *Stryphnus*^p^ *raratriaenus* |
| **Family Pachastrellidae** |  |  |
| Characella pachastrelloides (Carter, 1876) | Characella pachastrelloides | Astrophorida^p^ pachastrelloides |
| Dercitus bucklandi (Bowerbank, 1858) | Dercitus bucklandi | Ancorinidae^p^ bucklandi |
| *Pachastrella* sp. | *Pachastrella* sp. | *Pachastrella*^p^ sp. |
| Pachastrella ovisternata von Lendenfeld, 1907 | Pachastrella ovisternata | Pachastrella^p^ ovisternata |
| Poecillastra amygdaloides (Carter, 1876) | Nethea amygdaloides | Astrophorida^p^ amygdaloides |
| Poecillastra compressa (Bowerbank, 1866) | Poecillastra compressa | Vulcanellidae^p^ compressa |
| *Thenea abyssorum* Koltun, 1964 | *Thenea abyssorum* | *Thenea*^p^ *abyssorum* |
| *Thenea levis* von Lendenfeld, 1907 | *Thenea levis* | *Thenea*^p^ *levis* |
| *Thenea muricata* (Bowerbank, 1858) | *Thenea muricata* | *Thenea*^p^ *muricata* |
| *Thenea schmidti Sollas, 1886* | *Thenea schmidti* | *Thenea*^p^ *schmidti* |
| *Thenea valdiviae* von Lendenfeld, 1907 | *Thenea valdiviae* | *Thenea*^p^ *valdiviae* |
| *Triptolemma intextum* (Carter, 1876) | *Triptolemma intextum* | *Pachastrella*^p^ *intextum* |
| *Vulcanella (Annulastrella) ornata* (Sollas, 1888) | *Annulastrella ornata* | *Astrophorida*^p^ *ornata* |
| *Vulcanella (Vulcanella) aberrans* (Maldonado & Uriz, 1996) | *Vulcanella aberrans* | *Vulcanellidae*^p^ *aberrans* |
| *Vulcanella (Vulcanella) gracilis* (Sollas, 1888) | *Vulcanella gracilis* | *Vulcanellidae*^p^ *gracilis* |
| **Family Thrombidae** | **Family Thrombidae** |  |
| *Thrombus abyssi* (Carter, 1873) | *Thrombus abyssi* | *Astrophorida*^p^ *abyssi* |
| **Family Theonellidae** | **Family Theonellidae** |  |
| *Discodermia polymorpha* Pisera & Vacelet, in press | *Discodermia polymorpha* | *Theonellidae*^p^ *polymorpha* |
| *Theonella conica* (Kieschnick, 1896) | *Theonella conica* | *Theonellidae*^p^ *conica* |
| *Theonella swinhoei* Gray, 1868 | *Theonella swinhoei* | *Theonellidae*^p^ *swinhoei* |
| **Family Corallistidae** | **Family Corallistidae** |  |
| *Neophrissospongia nolitangere* (Schmidt, 1870) | *Neophrissospongia nolitangere* | *Astrophorida*^p^ *nolitangere* |
| **Family Phymaraphiniidae** | **Family Phymaraphiniidae** |  |
| *Exsuperantia* sp. | *Exsuperantia* sp. | *Astrophorida*^p^ sp. |
| **Family Alectonidae** |  |  |
| *Alectona millari* Carter, 1879 | *Alectona millari* | *Astrophorida*^p^ *millari* |
| *Neamphius huxleyi* (Sollas, 1888) | *Neamphius huxleyi* | *Astrophorida*^p^ *huxleyi* |
| **Outgroups** |  |  |
| **Spirophorida - Tetillidae** | **Spirophorida - Tetillidae** |  |
| *Craniella cranium* (Müller, 1776) | *Craniella cranium* |  |
| *Craniella* sp. | *Craniella* sp. |  |
| *Cinachyrella apion* (Uliczka, 1929) | *Cinachyrella apion* |  |
| Cinachyrella cf. schulzei (Keller, 1891) | Cinachyrella cf. schulzei |  |

* detected homonyms in the revised Linnaean classification that need to be addressed if these phylogenetic relationships are confirmed.
