# Supplementary material for: Molecular Phylogeny of the Astrophorida (Porifera, Demospongiae p) Reveals an Unexpected High Level of Spicule Homoplasy
Source: PLoS One. 2011 Apr 8;6(4):e18318. doi: 10.1371/journal.pone.0018318 (PMC3072971; doi:10.1371/journal.pone.0018318)
Supplement: File S1 — Definition of new clades defined in this study (following the rules of the PhyloCode v.4c). (DOC) [file pone.0018318.s008.doc]

**File S1**

Definition of new clades defined in this study (following the rules of the *PhyloCode v.4c* ([http://www.ohiou.edu/*PhyloCode*/](http://www.ohiou.edu/phylocode/)). For previously defined *Geodiidae*^p^ clade names used in this study see Cárdenas et al. (2010) [35].

- Ancorinidae Schmidt, 1870 (*nomen cladi conversum*): (branch-based) the most inclusive clade containing *Dercitus bucklandi* (Bowerbank, 1858) and not *Neamphius huxleyi* (Sollas, 1888) and *Geodia barretti* Bowerbank, 1858.
- Astrophorida Sollas, 1888 (*nomen cladi conversum*): (node-based) the least inclusive clade containing *Thrombus abyssi* (Carter, 1873) and *Pachymatisma johnstonia* (Bowerbank *in* Johnston, 1842).
- *Calthropella* von Lendenfeld, 1903 (*nomen cladi conversum*): (branch-based) the most inclusive clade containing *Calthropella geodioides* (Carter, 1876) and not *Caminella intuta* (Topsent, 1892).
- *Dragmastra* Sollas, 1888 (*nomen cladi conversum*): (branch-based) the most inclusive clade containing *Stelletta normani* Sollas, 1880 and not *Stelletta clarella* de Laubenfels, 1930.
- *Erylus* Gray, 1867 (*nomen cladi conversum*): (branch-based) the most inclusive clade containing *Erylus mamillaris* (Schmidt, 1862) and not *Pachymatisma johnstonia* (Bowerbank *in* Johnston, 1842).
- *Geostelletta* (*nomen cladi novum*): (branch-based) the most inclusive clade containing *Geodia tuberosa* (Topsent, 1892) and not *Geodia phlegraei* Sollas, 1880 and *Geodia angulata* von Lendenfeld, 1910.
- *Pachastrella* Schmidt, 1868 (*nomen cladi conversum*): (branch-based) the most inclusive clade containing *Pachastrella ovisternata* von Lendenfeld, 1894 and not *Nethea amygdaloides* (Carter, 1876) and *Pachymatisma johnstonia* (Bowerbank *in* Johnston, 1842) and *Theonella swinhoei* Gray, 1868.
- *Penares* Gray, 1867 (*nomen cladi conversum*): (branch-based) the most inclusive clade containing *Penares helleri* (Schmidt, 1864) and not *Erylus mamillaris* (Schmidt, 1862).
- *Stelletta* Schmidt, 1862 (*nomen cladi conversum*): (branch-based) the most inclusive clade containing *Stelletta grubii* Schmidt, 1862 and not *Stelletta normani* Sollas, 1880 and *Stryphnus mucronatus* (Schmidt, 1868).
- *Stryphnus* Sollas, 1886 (*nomen cladi conversum*): (branch-based) the most inclusive clade containing *Stryphnus mucronatus* (Schmidt, 1868) and not *Dercitus bucklandi* (Bowerbank, 1858).
- *Synops* Vosmaer, 1882 (*nomen cladi conversum*): (branch-based) the most inclusive clade containing *Geodia pachydermata* Sollas, 1886 *sensu* Topsent (1892) and not *Geodia gibberosa* Lamarck, 1815.
- *Thenea* Gray, 1867 (*nomen cladi conversum*): (branch-based) the most inclusive clade containing *Thenea muricata* (Bowerbank, 1858) and not *Annulastrella ornata* (Sollas, 1888).
- Theonellidae von Lendenfeld, 1903 (*nomen cladi conversum*): (branch-based) the most inclusive clade containing *Theonella swinhoei* Gray, 1868 and not *Characella pachastrelloides* (Carter, 1876).
- Vulcanellidae fam. nov. (*nomen cladi conversum*): (branch-based) the most inclusive clade containing *Poecillastra compressa* (Bowerbank, 1866) and not *Pachastrella ovisternata* von Lendenfeld, 1894.
