# Supplementary material for: Molecular Phylogeny of the Astrophorida (Porifera, Demospongiae p) Reveals an Unexpected High Level of Spicule Homoplasy
Source: PLoS One. 2011 Apr 8;6(4):e18318. doi: 10.1371/journal.pone.0018318 (PMC3072971; doi:10.1371/journal.pone.0018318)
Supplement: File S2 — Proposal for a new Linnaean classification of the Astrophorida. (DOC) [file pone.0018318.s009.doc]

**File S2**

Proposal for a revised Linnaean classification of the Astrophorida. New definitions have been given to well supported groups whose content has been modified by our study. The lithistids families are not detailed since no taxonomical modifications have been made within them.

Order Astrophorida Sollas, 1888

**Family Thrombidae Sollas, 1888**

*Thrombus* Sollas, 1886

*Yucatania* Gómez, 2006

**Family Thoosidae Rosell and Uriz, 1997** (resurrected)

*Alectona* Carter, 1879

*Delectona* de Laubenfels, 1936

*Thoosa* Hancock, 1849

**Family Theneidae Carter, 1883** (resurrected)

New definition: Astrophorida with long-shafted triaenes (sometimes lost) in combination with diverse categories of streptasters: spirasters, metasters and plesiasters (sometimes with annulate actines).

*Annulastrella* Maldonado, 2002

*Cladothenea* Koltun, 1964

*Thenea* Gray, 1867

*Lamellomorpha* Bergquist, 1968 *incertae sedis*

*Characella* Sollas, 1886 *incertae sedis*

New Definition: Astrophorida with a majority of amphiasters as streptasters (never spirasters) and with at least two clearly separated categories of monaxonic spicules: the longest (microxea, microstyles, microstongyloxeas) and the smallest category (microrhabds with oxea or strongyle ends).

*Neamphius* de Laubenfels, 1953 *incertae sedis*

*Acanthotriaena* Vacelet et al., 1976 *incertae sedis*

*Ancorella* von Lendenfeld, 1907 *incertae sedis*

*Jaspis Gray, 1867 incertae sedis*

*Holoxea* Topsent, 1892 *incertae sedis*

**Family Corallistidae Sollas, 1888**

**Family Isoraphiniidae Schrammen, 1924**

**Family Macandrewiidae Schrammen, 1924**

**Family Neopeltidae Sollas, 1888**

**Family Phymaraphiniidae Schrammen, 1924**

**Family Phymatellidae Schrammen, 1910**

**Family Pleromidae Sollas, 1888**

**Family Theonellidae von Lendenfeld, 1903**

**Family Pachastrellidae Carter, 1875**

New definition: Astrophorida with a majority of amphiasters as streptasters (never spirasters) in combination with large calthrops and/or short-shafted mesotriaenes or mesotrider desmas. A variety of monaxonic spicules can be present: microxeas, microrhabds, microstrongyles and microrhabdose streptasters.

*Brachiaster* Wilson, 1925

*Nethea* Sollas, 1888 (resurrected)

New Definition: Pachastrellidae with triactinal calthrops (or dichocalthrops).

*Pachastrella* Schmidt, 1868

*Triptolemma* de Laubenfels, 1955

**Family Vulcanellidae fam. nov.**Definition: Astrophorida with calthrops, short-shafted triaenes or long-shafted triaenes. Aster microscleres include several categories of streptasters (spirasters, metasters, amphiasters and plesiasters). Monaxonic spicules consist of one to three categories of spiny microxeas.

Type species of the family: *Vulcanella gracilis* (Sollas, 1888).

*Poecillastra* Sollas, 1888

New Definition: Vulcanellidae with spiny microxeas in a single category, triaenes are pseudocalthrops and/or short-shafted triaenes.

*Vulcanella* Sollas, 1886

New Definition: Vulcanellidae with spiny microxeas in one to three categories, with a more or less conspicuous ringed ornamentation, triaenes are calthrops and/or short-shafted triaenes or long-shafted triaenes.

**Family Geodiidae Gray, 1867**

New definition: Astrophorida with microrhabds, spherules and a diversity of euasters. Sterrasters is a synapomorphy of this group although they have been secondarily lost in some taxa.

- Subfamily Erylinae Sollas, 1888
  - *Caminus* Schmidt, 1862
  - ?*Melophlus* Thiele, 1899
  - *Erylus* Gray, 1867
  - *Pachymatisma* Bowerbank *in* Johnston, 1842
  - *Penares* Gray, 1867
- Subfamily Geodinae Sollas, 1888
  - *Geodia* Lamarck, 1815
    New definition: Geodiidae with euasters or large microrhabds in the ectocortex and sterrasters in the endocortex (sometimes secondarily lost).
- Subfamily Calthropellinae von Lendenfeld, 1907
  - *Calthropella* Sollas, 1888
  - *Corticellopsis* Bergquist, 1968
  - *Pachataxa* de Laubenfels, 1936

*Caminella* von Lendenfeld, 1894 (resurrected)

Definition: Geodiidae with uniporal oscule leading into a cloaca. Ectocortex microscleres are spherasters and/or strongylasters, endocortex microscleres are globular sterrasters.
Type species: *Caminella intuta* (Topsent, 1892)

**Family Ancorinidae Schmidt, 1870**

- Subfamily Sanidasterinae Sollas, 1888 (resurrected)
  - *Dercitus* Gray, 1867
    - *Dercitus (Dercitus)* Gray, 1867
    - *Dercitus (Halinastra)* de Laubenfels, 1936
    - *Dercitus (Stoeba)* Sollas, 1888
  - *Disyringa* Sollas, 1888
  - ?*Ecionemia* Bowerbank, 1864 (in part).
  - ?*Psammastra* Sollas, 1886
  - *Stryphnus* Sollas, 1886
  - ?*Tribrachium* Weltner, 1882
- Subfamily Stellettinae Carter, 1875 (resurrected)
  - ?*Ancorina* Schmidt, 1862
  - ?*Cryptosyringa* Vacelet, 1979
  - *Stelletta* Schmidt, 1862
  - *Tethyopsis* Stewart, 1870
