# Supplementary material for: Molecular Phylogeny of the Astrophorida (Porifera, Demospongiae p) Reveals an Unexpected High Level of Spicule Homoplasy
Source: PLoS One. 2011 Apr 8;6(4):e18318. doi: 10.1371/journal.pone.0018318 (PMC3072971; doi:10.1371/journal.pone.0018318)
Supplement: File S3 — Key to the Astrophorida families, sub-families and genera incertae sedis. (DOC) [file pone.0018318.s010.doc]

**File S3**

Key to the Astrophorida families, sub-families and genera *incertae sedis*. The part of the key leading to the lithistid families is modified from Pisera & Lévi (2002) [96].

1. Desmas absent 2
   Desmas present 14
2. Asters absent 3
   Asters present 4
3. Microscleres are spiny microxeas; calthrops with a reduced fourth actine *Ancorella*
   Microrhabds in ectocortex; short-shafted triaene **Geodiidae** (Erylinae), *Penares* (in part)
   Microscleres trichotriaenes are present **Thrombidae**, *Thrombus* (in part)
   Microscleres include sanidasters; short-shafted dichotriaenes **Ancorinidae** (Sanidasterinae), *Stoeba*
   Microscleres include sanidasters and trichodragmata (sometimes lost); triaenes absent *Holoxea*
   No microscleres; long-shafted plagiotriaenes; anatriaenes and oxeas *Stelletta anasteria*
4. Asters are euasters (sterrasters, oxyasters, spherasters, strongylasters,…), sometimes modified to toxas 5
   Asters are streptasters (plesiasters, metasters, spirasters and/or amphiasters) 6
   Asters are amphiasters and thin-rayed oxyasters (sometimes modified to toxas); excavating sponge
    **Thoosidae**, *Thoosa*
5. Microscleres include sanidasters; triaenes are long-shafted triaenes (sometimes secondarily lost) or calthrops **Ancorinidae** (Sanidasterinae)^1^
   Euasters include sterrasters (sometimes secondarily lost*) in the endocortex and another kind of euasters in the ectocortex; triaenes are long-shafted 7
   Euasters include sterrasters or aspidasters (sometimes secondarily lost*) in the endocortex and spherules or microrhabds in the ectocortex; triaenes are short-shafted; ana/pro/mesotriaenes are absent **Geodiidae** (Erylinae)
   Microscleres do not include sterrasters/aspidasters and sanidasters 8
6. Streptasters are mainly amphiasters 9
   Streptasters are mainly spirasters and plesiasters 10
7. Uniporal oscule leads into an atrium **Geodiidae** (*Caminella*)
   Uniporal oscule does not lead into an atrium **Geodiidae** (Geodinae)
8. Triaenes are long-shafted **Ancorinidae** (Stellettinae)
   Triaenes are calthrops and short-shafted mesotriaenes **Geodiidae** (Calthropellinae)
   Triaenes are absent *Jaspis*
9. Megascleres include calthrops (sometimes with a reduced fourth actine) or short-shafted mesotriaenes and/or mesotrider desmas **Pachastrellidae**
   Megascleres are long-shafted triaenes *Characella*
   Triaenes are absent 11
10. Microxeas present; no acanthotriaenes **Vulcanellidae**
    Microxeas present; long-shafted acanthotriaenes present *Acanthotriaena*
    Microxeas absent **Theneidae**
11. Robust diactine or polyactine megascleres; excavating sponge **Thoosidae,** *Alectona*
    Robust diactine/polyactine megascleres absent 12
12. Trichotriaenes present **Thrombidae**
    Trichotriaenes absent 13
13. No megascleres; microscleres include microrhabds **Thoosidae**, *Delectona*
    Megascleres include only oxeas; excavating sponge in its early stage *Neamphius*
    Megascleres include oxeas, strongyles and strongyloxeas; other microscleres are microstrongyles
     *Lamellomorpha*
14. Desmas as megaclones; ectosomal dichotriaenes (and sometimes anatriaenes) **Pleromidae**
    Desmas as heloclones; ectosomal plagio and/or dichotriaenes **Isoraphiniidae**
    Desmas as dicranoclones; ectosomal dichotriaenes **Corallistidae**
    Desmas as triders; ectosomal discotriaenes to phyllotriaenes **Phymaraphiniidae**
    Desmas with triaenose, rarely monoaxial crepis; ectosomal discotriaenes to phyllotriaenes; asters are absent **Macandrewiidae**
    Desmas as tetraclones 15
    Desmas of complex branching shape; monoaxial ectosomal phyllotriaenes or discotriaenes **Neopeltidae**
15. Ectosomal dichotriaenes **Phymatellidae**
    Ectosomal discotriaenes to phyllotriaenes; asters are sometimes absent **Theonellidae**

* DNA sequencing and a molecular phylogenetic analyses is necessary to reveal this loss.

^1^ The phylogenetic position of *Ancorina* is ambiguous (Figure 2). To simplify this key, we considered *Ancorina* to be part of the Sanidasterinae (as suggested by morphological data).
